# Comprehensive establishment and characterization of orthoxenograft mouse models of malignant peripheral nerve sheath tumors for personalized medicine

**DOI:** 10.15252/emmm.201404430

**Published:** 2015-03-25

**Authors:** Joan Castellsagué, Bernat Gel, Juana Fernández-Rodríguez, Roger Llatjós, Ignacio Blanco, Yolanda Benavente, Diana Pérez-Sidelnikova, Javier García-del Muro, Joan Maria Viñals, August Vidal, Rafael Valdés-Mas, Ernest Terribas, Adriana López-Doriga, Miguel Angel Pujana, Gabriel Capellá, Xose S Puente, Eduard Serra, Alberto Villanueva, Conxi Lázaro

**Affiliations:** 1 Hereditary Cancer Program, Catalan Institute of Oncology (ICO-IDIBELL), L'Hospitalet de LlobregatBarcelona, Spain; 2 Translational Research Laboratory ICO-IDIBELL, L'Hospitalet de LlobregatBarcelona, Spain; 3 Institut de Medicina Predictiva i Personalitzada del Càncer (IMPPC)Badalona, Barcelona, Spain; 4 Pathology Service, HUB-IDIBELL, L'Hospitalet de LlobregatBarcelona, Spain; 5 Unit of Infections and Cancer (UNIC), Cancer Epidemiology Research Program ICO-IDIBELL and CIBER Epidemiología y Salud Pública (CIBERESP), L'Hospitalet de LlobregatBarcelona, Spain; 6 Plastic Surgery Service HUB-IDIBELL, L'Hospitalet de LlobregatBarcelona, Spain; 7 Medical Oncology Service ICO-IDIBELL, L'Hospitalet de LlobregatBarcelona, Spain; 8 Instituto Universitario de Oncología del Principado de Asturias (IUOPA), Universidad de OviedoOviedo, Spain

**Keywords:** MPNST, NF1, patient-derived tumor xenograft, preclinical mouse models, sorafenib

## Abstract

Malignant peripheral nerve sheath tumors (MPNSTs) are soft-tissue sarcomas that can arise either sporadically or in association with neurofibromatosis type 1 (NF1). These aggressive malignancies confer poor survival, with no effective therapy available. We present the generation and characterization of five distinct MPNST orthoxenograft models for preclinical testing and personalized medicine. Four of the models are patient-derived tumor xenografts (PDTX), two independent MPNSTs from the same NF1 patient and two from different sporadic patients. The fifth model is an orthoxenograft derived from an NF1-related MPNST cell line. All MPNST orthoxenografts were generated by tumor implantation, or cell line injection, next to the sciatic nerve of nude mice, and were perpetuated by 7–10 mouse-to-mouse passages. The models reliably recapitulate the histopathological properties of their parental primary tumors. They also mimic distal dissemination properties in mice. Human stroma was rapidly lost after MPNST engraftment and replaced by murine stroma, which facilitated genomic tumor characterization. Compatible with an origin in a catastrophic event and subsequent genome stabilization, MPNST contained highly altered genomes that remained remarkably stable in orthoxenograft establishment and along passages. Mutational frequency and type of somatic point mutations were highly variable among the different MPNSTs modeled, but very consistent when comparing primary tumors with matched orthoxenografts generated. Unsupervised cluster analysis and principal component analysis (PCA) using an MPNST expression signature of ~1,000 genes grouped together all primary tumor–orthoxenograft pairs. Our work points to differences in the engraftment process of primary tumors compared with the engraftment of established cell lines. Following standardization and extensive characterization and validation, the orthoxenograft models were used for initial preclinical drug testing. Sorafenib (a BRAF inhibitor), in combination with doxorubicin or rapamycin, was found to be the most effective treatment for reducing MPNST growth. The development of genomically well-characterized preclinical models for MPNST allowed the evaluation of novel therapeutic strategies for personalized medicine.

## Introduction

Malignant peripheral nerve sheath tumors (MPNSTs) are rare malignancies with a peripheral nerve sheath origin. MPNSTs account for 3–10% of all soft tissue sarcomas and are a highly aggressive histological subtype, with an incidence in the general population of 1 per 100,000 (Ducatman *et al*, 1986; Collin *et al*, 1987; Evans *et al*, 2002). Approximately half of MPNSTs develop in patients with neurofibromatosis type 1 (NF1), while the other half develop sporadically (Evans *et al*, 2002; Ferner & Gutmann, 2002; Carli *et al*, 2005). NF1 is a common autosomal dominant tumor predisposition syndrome occurring in 1 in 3,500 individuals world-wide (Huson *et al*, 1989; Evans *et al*, 2002). NF1 patients develop benign dermal and plexiform neurofibromas and MPNSTs. Inactivation of the remaining *NF1* wild-type allele is essential for neurofibroma formation, although genetically engineered mouse models have shown that a heterozygous *NF1* (+/−) cell environment is important for its development (Zhu *et al*, 2002). In NF1, MPNSTs commonly arise within a preexisting plexiform neurofibroma (Ducatman *et al*, 1986). The lifetime risk of MPNST development in NF1 patients is around 8–13%, and these sarcomas are the leading cause of mortality and morbidity in adults with NF1 (Rasmussen *et al*, 2001; Evans *et al*, 2002). Due to the disease progression and metastatic potential, both sporadic and NF1-related MPNSTs are considered tumors of poor prognosis (Ferner & Gutmann, 2002; Porter *et al*, 2009).

The therapeutic approach for all MPNSTs comprises surgical excision followed by radiation and/or chemotherapy (Ducatman *et al*, 1986; Carli *et al*, 2005; Dilworth *et al*, 2006; Porter *et al*, 2009; Moretti *et al*, 2011). The 5-year survival rate after MPNST diagnosis in a NF1 patient is 20–50%, with a higher survival rate in sporadic cases (Evans *et al*, 2002). Treatment failure is often associated with bone and lung metastases (Ducatman *et al*, 1986; Wong *et al*, 1998; Anghileri *et al*, 2006). Standard sarcoma chemotherapy regimens are indicated for the treatment of MPNSTs.

Different strategies have been developed to generate *in vivo* tumor models that may resemble human MPNST and could be used to assess effective, standardized therapies. Subcutaneous and orthotopic xenograft MPNST models have been generated from both sporadic (Mahller *et al*, 2007; Johansson *et al*, 2008; Lopez *et al*, 2011) and NF1 tumors, from established cancer cell lines in all cases (Perrin *et al*, 2007; Banerjee *et al*, 2010; Lopez *et al*, 2011; Turk *et al*, 2011). To date, only one model has been derived from a primary MPNSTs, but this was subcutaneously engrafted (Bhola *et al*, 2010). A genetically engineered mouse model carrying linked germline mutations in *Nf1* and *Tp53* or *Pten* has also been developed and used in several drug trials (Cichowski *et al*, 1999; Vogel *et al*, 1999; Keng *et al*, 2012).

Several therapeutic approaches have been evaluated in preclinical models (Killion *et al*, 1998; Mahller *et al*, 2007; Ambrosini *et al*, 2008; Johansson *et al*, 2008; Demestre *et al*, 2010; Jessen *et al*, 2013; Ohishi *et al*, 2013), most of which target the RAS-MAPK signaling pathway and the mTOR pathway (Basu *et al*, 1992; DeClue *et al*, 1992; Guha *et al*, 1996; Downward, 2003; Watson *et al*, 2014), which is expected to be over-activated upon *NF1* mutation (Guha *et al*, 1996; Sherman *et al*, 2000). However, the results of these assays are inconclusive or limited to certain models. Two recent phase II clinical trials assessed the monotherapy activity of sorafenib or rapamycin analogs (temsirolimus) in patients with different types of sarcoma, including MPNSTs (Maki *et al*, 2009; Okuno *et al*, 2011). In general, no objective responses were observed in this subset of patients when using a single drug treatment.

Here, we describe the establishment and comprehensive characterization of a library of orthotopic patient-derived xenograft MPNST models from sporadic and NF1 patients. Our results demonstrate that perpetuated orthotopic patient-derived tumor xenografts (PDTXs) closely resemble primary tumors and allow preclinical evaluation of personalized therapeutic approaches.

## Results

### Development of orthoxenograft mouse models of MPNSTs

**Figure 1:**
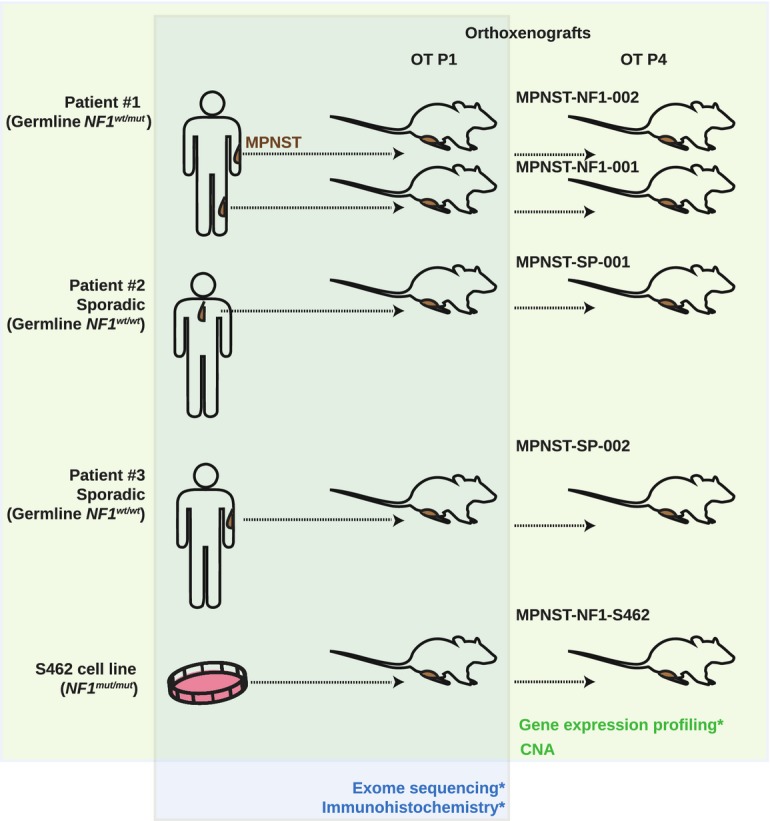
Development of orthoxenograft mouse models of MPNSTs Five orthotopic xenograft mice models were established from four different MPNSTs and one cell line. Two of the MPNSTs come from the same NF1 patient; the other two were sporadic cases. Primary tumor (2–3 mm^3^) or tumor cell line (3 × 10^6^ cells) were grafted or injected in the leg of athymic nude mice, close to the sciatic nerve. Tumors were perpetuated along several passages and subsequently expanded. Several assays were performed on tumors in early passages (histopathological analysis, gene expression profiling, genomic profiling, and drug efficacy studies). *NF1-MPNST-001 at passage 4 (P4) and all primary tumors and orthotopic tumors (OT) at passage 1 (P1) were analyzed by exome sequencing and immunohistochemistry. All primary tumors and orthotopic tumors at passages 1 and 4 were analyzed by expression array (except NF1-MPNST-001 PT, SP-MPNST-001 OT P4, and NF1-S462 OT P4). Copy number analysis (CNA) was performed in all samples.

We generated five MPNST orthoxenograft mouse models: two from sporadic tumors, two from independent tumors of the same NF1 patient, and one corresponding to the engraftment of the MPNST cell line S462 (Fig1 and Table1). None of the primary tumors received radiotherapy or chemotherapy prior to surgery. Human tumors (2–3 mm^3^) were grafted onto the sciatic nerve of nude mice following the procedure outlined in Materials and Methods.

**Table 1:** Clinical characteristics of patients and tumors used to generate the xenograft models

Tumor ID	Age	Sex	Ethnicity	NF1 patient	Germline *NF1*	MPNST	Tumor grade	Location	Somatic *NF1*
MPNST-NF1-001	34	M	EU (Spain)	Yes	c.350T>A	Primary	IV	Thigh	LOH
MPNST-NF1-002	37	M	EU (Spain)	Yes	c.350T>A	Primary	III	Arm	LOH
MPNST-SP-001	88	M	EU (Spain)	No	–	Primary	IV	Laterocervical	c.3520C>T
MPSNT-SP-002	74	F	EU (Italy)	No	–	Relapse	IV	Arm	–
MPNST-NF1-S462	19	F	EU? (Germany?)	Yes	c.6792C>A	Cell line	IV	Thigh	LOH

EU, European.

### Orthoxenograft mouse MPNST models closely resemble primary tumors

Having established and standardized the orthoxenograft models, we performed an exhaustive histological and molecular characterization of MPNSTs and orthoxenografts, comparing each primary tumor to its corresponding orthoxenograft at passages 1 and 4.

#### Histological validation

**Figure 2:**
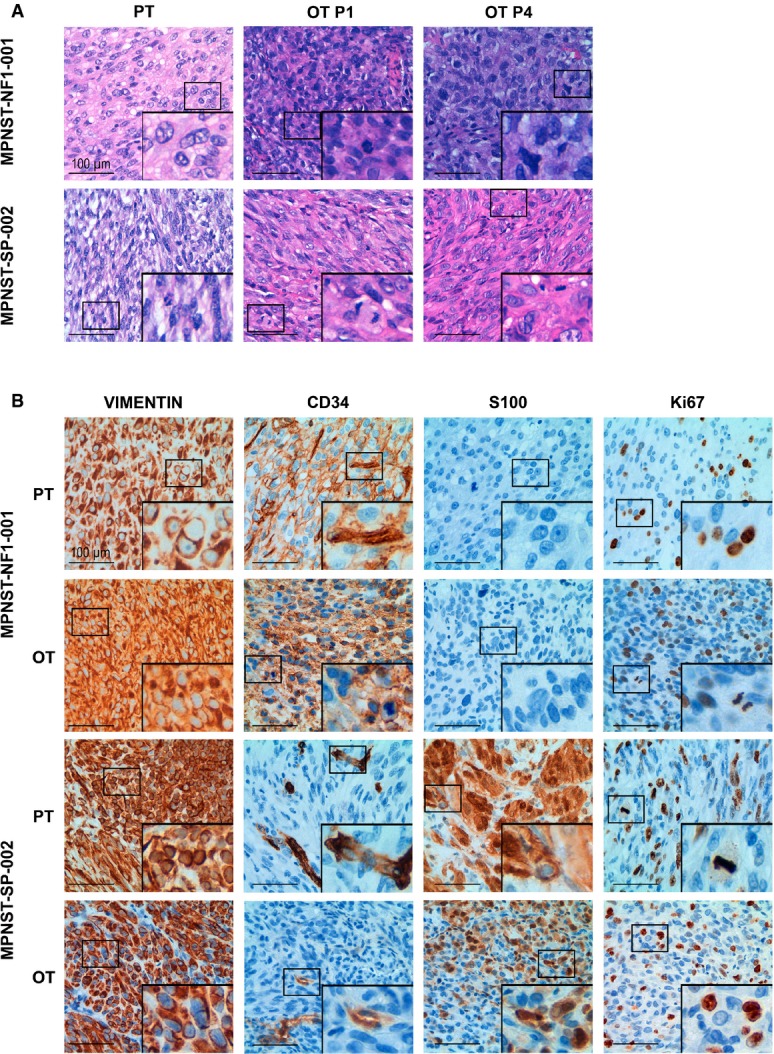
Orthoxenograft mouse MPNST models closely resemble primary tumors

Hematoxylin–eosin staining showed similar histopathological patterns between primary tumors and orthoxenografts at passages 1 and 4 (Fig2A and Supplementary Fig S1A). In addition, analysis of the soft-tissue tumor marker vimentin showed positivity in all models, while three canonical non-nerve tumor markers (epithelial membrane antigen, desmin, and smooth muscle actin) were all negative (Table2, Fig2B and Supplementary Fig S1B). The endothelial marker CD34 was shown to be positive in two of the NF1 tumor models (including the corresponding primary tumors) but negative for the rest of cases. S100, a neural differentiation marker that stains all benign Schwann cell tumors but only ~50% of MPNSTs (Khalifa *et al*, 2000), revealed positivity in all sporadic models but was negative for the NF1 tumors. As measured by Ki-67 staining, the rate of tumor cell proliferation was similar for all cases (positivity 25–35%) with the exception of S462 cells, which showed a higher proliferation rate (~80%); similar results were observed using P53 staining (Table2, Fig2B and Supplementary Fig S1B).

**Table 2:** Immunohistochemical characterization of human tumors and their first derived xenograft mouse models

Antibody	MPNST-NF1-001		MPNST-NF1-002		MPNST-SP-001		MPNST-SP-002		MPSNT-NF1-S462	
	PT	OT	PT	OT	PT	OT	PT	OT	CL	OT
Vimentin	+	+	+	+	+	+	+	+	+	+
Desmin	−	−	−	−	−	−	−	−	−	−
Actin	−	−	−	−	−	−	−	− (+ focal)	−	− (+ focal)
EMA	−	−	−	−	−	−	−	−	−	−
CD34	+	+	−	− (+ focal)	−	−	−	−	+	+
S100	−	−	−	−	+	+	+	+/−	−	−
P53	15%	15%	<5%	20%	<5%	<5%	20%	<5%	80%	80%
Ki-67	25%	25%	25–30%	35%	35–40%	20%	25–30%	25–30%	90–95%	80%

PT, primary tumor; OT, orthotopic xenograft tumor; CL, cell line.

Orthotopic MPNST xenografts at passages 1 (OT P1) and 4 (OT P4) were histopathologically similar to their corresponding primary MPNST (PT) in hematoxylin–eosin staining of paraffin-embedded tumor sections from patients MPNST-NF1-001 and MPNST-SP-002. Main panels show a general view of the tumors at low magnification (40×); inset pictures were taken at higher magnification (400×).

Orthotopic xenograft and primary MPNSTs exhibited similar immunohistochemical features. A representative immunostained section of vimentin, CD34, S100, and Ki-67 is shown for primary tumors (PT) and orthotopic tumors (OT) from patients MPNST-NF1-001 and MPNST-SP-002. Positive antibody signals are shown in brown, and the hematoxylin counterstain in blue. Main panels show pictures at high magnification (400×); inset pictures show mitotic cells present in these tumors.

To investigate distal dissemination properties, lung, brain, and liver from sacrificed mice were histologically analyzed for the presence of micrometastases. Synchronic micrometastases were identified in lung from three of the models (MPNST-SP-001, MPNST-SP-002, and the orthotopically engrafted S462 cell line) (Supplementary Fig S2), but no liver or brain metastases were identified. Moreover, to better characterize the metastatic phenotype, one of the synchronous micrometastases of MPNST-SP-002 was immunochemically characterized using four antibodies: vimentin, CD34, S100, and Ki-67, which were a perfect match with the corresponding orthoxenograft MPNSTs (Supplementary Fig S2).

A subgroup of orthotopically implanted mice (the two sporadic models and MPNST-NF1-001) was kept alive for 4–6 months after tumor removal to investigate the dissemination capabilities over a longer time frame. Metachronic micrometastases were only identified in the lung from one sporadic MPNST tumor (Supplementary Fig S2).

### Human stroma is lost after engraftment and replaced by murine cells

**Figure 3:**
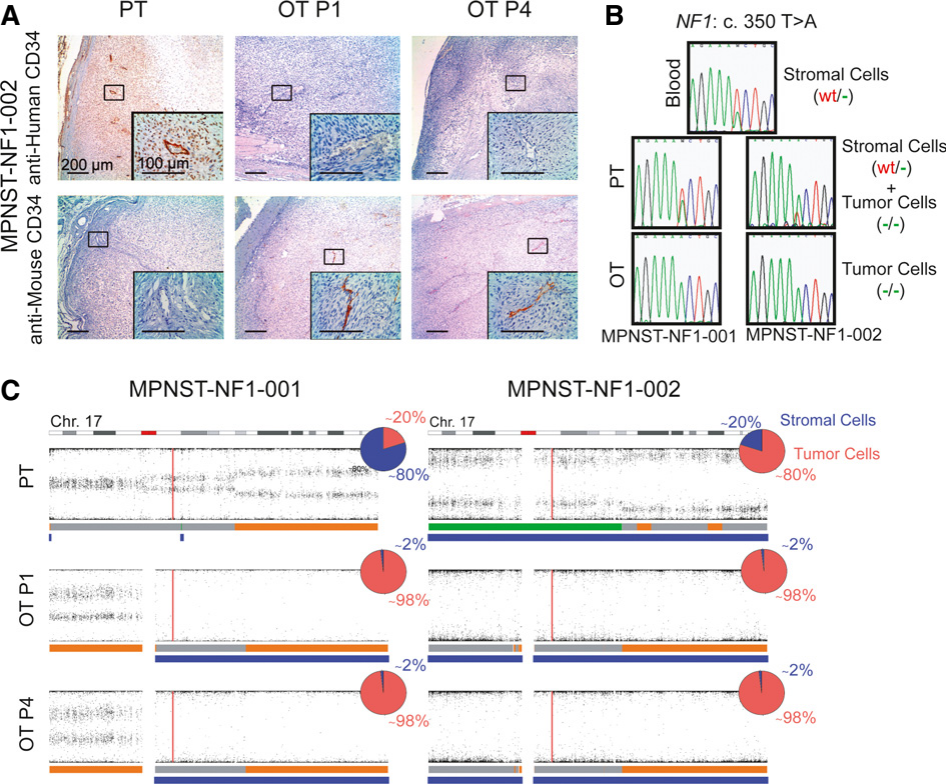
Human stroma is lost after engraftment and replaced by murine cells

Due to the importance of tumor microenvironment in tumor behavior and response to therapy, it was important to understand the nature of the stroma in the MPNST orthoxenografts generated. Thus, we next analyzed the fate of human non-tumor stromal cells after primary MPNST engraftment. Staining with anti-human CD34 clearly labeled vessels in primary tumors but not in orthoxenografts. By contrast, an antibody for the identification of mouse CD34 only labeled vessels in the orthoxenograft samples and not in the primary tumor (Fig3A and Supplementary Fig S3). In addition, when attempting to derive cell lines from first-passage MPNST orthoxenografts, a rapid overgrowth of murine fibroblasts was observed immediately after plating (data not shown), indicating the presence not only of murine vessels but also mouse stromal fibroblasts.

Stromal elements of the primary tumors were labeled with anti-human CD34 but not anti-mouse CD34; patient-derived xenografts are labeled with anti-mouse CD34 only and no anti-human marker. Representative sections (at 40× and 400× magnification) of the primary tumors (PT) and the orthoxenograft tumors at passages 1 (OT P1) and 4 (OT P4) were labeled with anti-human CD34 (H) and anti-mouse CD34 (M).

Sanger sequencing of the germline *NF1* mutation c.350T>A present in the patient that developed two different tumors (MPNST-NF1-001 and MPNST-NF1-002). Sequencing results from normal tissue, primary tumor (PT), and orthotopic xenograft tumors (OT) are shown. The sequence of the *NF1* region revealed WT *NF1* alleles in primary tumor samples, and not in the corresponding derived orthoxenografts, indicating probable loss of the human stromal cells.

SNP array of primary tumor (PT) and orthotopic xenograft passages 1 (OT P1) and 4 (OT P4) for MPNST-NF1-001 and MPNST-NF1-002 tumors. Results correspond to chromosome 17. Images show the B-allele frequency (BAF) for the different samples as scatter plots and the copy number callings below them represented by thick horizontal lines: 2n regions are shown in gray, gained regions in orange, lost regions in green, and LOH regions are represented in blue. The vertical red line indicates the location of the *NF1* locus. In addition, the percentage of tumor versus stromal cells for each sample is represented in a pie chart (blue, stromal cells; red, tumor cells).

As both copies of the *NF1* gene are inactivated in NF1-associated MPNST, non-malignant stromal cells can be identified as those carrying only the constitutional mutation but not bearing a second *NF1* hit. We analyzed the NF1 patient-derived MPNSTs (NF1-001 and NF1-002) for the presence of mutation c.350T>A (germline hit) and for the second *NF1* hit (LOH in both tumors). The sequence of the *NF1* region containing the constitutive mutation revealed WT *NF1* alleles in primary tumor samples, indicating the presence of normal human cells (Fig3B). By contrast, WT *NF1* alleles were cleared out in the corresponding derived orthoxenografts, indicating probable loss of the human stromal cells (Fig3B). We then analyzed SNP array data from both tumors and corresponding xenografts, using ASCAT to estimate the percentage of normal cells present in both sample types. These results corroborated the loss of human stroma cells in orthoxenografts from the first engraftment (passage 1) (Fig3C). Further analysis of SNP array data from sporadic MPNSTs showed a similar pattern of stromal loss.

#### Molecular validation

In addition to a thorough histological validation of the developed MPNST orthoxenograft models, we performed extensive molecular characterization at the genomic and transcriptomic levels of primary tumors and orthoxenografts by SNP array, exome sequencing, and expression array analyses.

### Genomic copy number and allelic imbalance analysis

MPNSTs are characteristically composed of tumor cells containing highly altered genomes at a structural level (Forus *et al*, 1995; Mertens *et al*, 1995, 2000; Mechtersheimer *et al*, 1999; Mantripragada *et al*, 2008, 2009; Beert *et al*, 2011). Accordingly, we characterized the somatic copy number alterations (SCNAs) and allelic imbalances (AIs) present in primary tumors and paired orthoxenografts and performed an exhaustive comparison.

**Figure 4:**
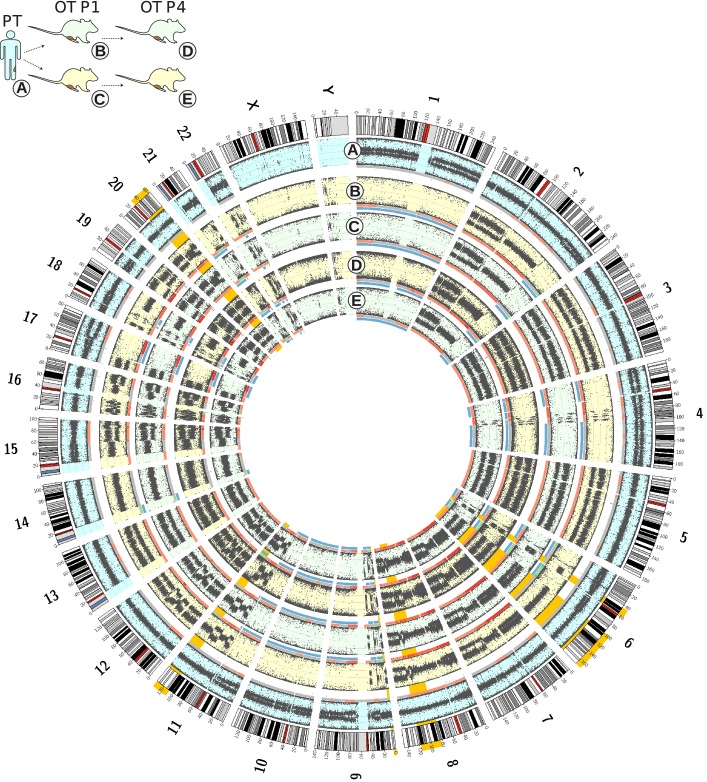
Orthotopic xenograft MPNSTs maintain the genomic structure found in primary tumors Genome-wide SNP array profiling from two different orthoxenograft tumors derived from the same primary tumor (MPNST-NF1-001) are shown as Circos plots. The outermost layer contains the set of canonical human chromosomes. The following layers, from outside to inside, illustrate the following: the BAF of the primary tumor (A), and the derived xenografts at passages 1 (B and C) and 4 (D and E). Copy number variations are represented by a colored line under each BAF (gray: 2n, red: > 2n (chromosomal gain); green: < 2n (chromosomal loss)). LOH events are shown in blue. Finally, differences between primary and xenograft tumors not compatible with the loss of signal from stroma cells are highlighted in orange.

We first analyzed tumor MPNST-NF1-001 by comparing the primary tumor with the orthoxenografts at passages 1 and 4 from two lineages representing two independent engraftments (Fig4). SNP array data from these five samples were analyzed using ASCAT. Comparison of the primary tumor with the four orthoxenografts allowed us to detect genomic alterations along xenograft passages, assessing the genomic stability of the engrafted tumor, and differences between two primary engrafted independent lineages, assessing the reproducibility of the orthoxenograft model. As expected, the genome of the primary MPNSTs and orthoxenografts was highly altered, mainly presenting gains of whole chromosomes or large chromosomal regions and a few losses of genetic material. In addition, B-allelle frequency (BAF) plots showed several patterns consistent with complex rearrangements and large regions exhibiting LOH (Fig4). A global view of the genomic alteration profiles showed a high degree of similarity between the primary tumor and the 4 derived orthoxenografts. In this case, due to the high proportion of non-altered stroma cells in the primary tumor sample, the raw data were strongly biased toward a diploid heterozygous genome; hence, the variant calling algorithm used reported fewer alterations in the primary tumor than in orthoxenografts. However, visual inspection of the raw data revealed that almost all alterations identified in orthoxenografts were present in primary tumors (see Fig4). Furthermore, these differences were not present in the rest of the primary tumor versus orthoxenograft comparisons, since these tumors contained a lower proportion of 2n cells (Supplementary Fig S4A and B). The comparison of BAF plots between primary tumor and orthoxenograft passages 1 and 4 was consistent with the progressive depletion of human 2n cells along passages. In addition, the analysis of multiple orthoxenograft passages revealed that this highly altered genome remained stable along successive xenograft passages (Supplementary Fig S4A and B). The differences in BAF between orthoxenografts at passage 1 and passage 4 that were not compatible with progressive stromal removal were interpreted as structural genomic changes caused by the successive engraftments (highlighted in Supplementary Fig S4A and B). Overall, comparative analysis of the primary tumor and the serial passages of the orthoxenograft models indicated that, on average, < 7% of the orthoxenograft genome presented structural changes (copy number alterations and allelic imbalances) relative to the primary tumor.

To overcome any bias produced by different proportions of 2n stromal cells in the primary tumors, we compared passage 1 orthotopic xenografts, in which the human stroma was strongly reduced (Fig3 and Supplementary Fig S3). Both the sporadic and NF1-related tumors presented a highly altered genome with several copy number alterations, ranging from 215 in MPNST-NF1-001 to 401 in MPNST-NF1-002, affecting the majority of the genome (from 71% in MPNST-NF1-001 to 84.9% in MPNST-SP-002). LOH was of 38.3 and 61.2% in NF1-related tumors (MPNST-NF1-001 and MPNST-NF1-002) and of 3.8 and 24% in sporadic cases (MPNST-SP-001 and MPNST-SP-002, respectively) (Supplementary Fig S4A and B).

### Exome sequencing

Exome sequencing was used to characterize and compare genetic variation caused by point mutations in genomic coding regions present in constitutional DNA, primary tumors, the S462 cell line, and matched orthoxenograft MPNST models. The minimum coverage needed for reliable variant calling was set at 20×, and regions above this threshold (well covered regions, WCR) were identified in all samples related to the same primary tumor (primary related samples, PRS). The length of the different PRS-WCR ranged from 16.9 Mb for MPNST-NF1-001-PRS to 44 Mb for MPNST-SP-001-PRS. Only variants present in PRS-WCR were taken into account when comparing samples within each PRS (Supplementary Table S1).

**Figure 5:**
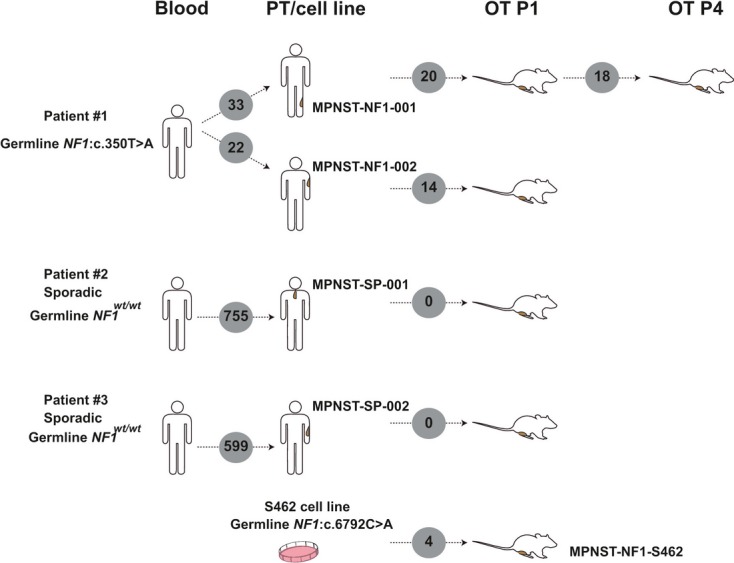
Exome-sequencing analysis Number of somatic point mutations identified in primary tumors and new mutations acquired in the orthoxenograft models in PRS-WCR. Somatic mutations found in primary tumors in sporadic cases were maintained in the orthoxenograft-derived tumors, whereas few acquired mutations were observed in all NF1-derived models. PT, primary tumor; OT, orthotopic xenograft tumor; passage 1 (P1); passage 4 (P4).

The number of somatic mutations identified in PRS-WCR varied between PRSs and ranged from 22 in MPNST-NF1-002-PRS to 755 in MPNST-SP-001-PRS. When comparing tumors and xenograft pairs at passage 1, just after engraftment, a mean of ~9 (0–33) mutations was identified in orthoxenografts that were not present in primary tumors (Fig5) (Supplementary Table S1). New mutations were scattered over the genome and showed no apparent clustering except for three intronic mutations in the *TTN* gene. Of a total of 1,409 somatic mutations identified in the four primary tumors, only 6 (3 in MPNST-NF1-002-OT1 and 3 in MPNST-NF1-001-OT4) were not detected in the orthotopic xenograft models and therefore classified as lost in the engraftment process (data not shown). Altogether, the low number of new and lost point mutations detected in the engrafted tumors with respect to their primary counterparts reinforces our observation that the orthotopic xenograft MPNSTs generated for this study recapitulate the characteristics of the primary tumors.

A striking difference in the number and type of somatic point mutations was observed when comparing *NF1*-derived and sporadic models. In order to remove any bias due to different coverage depths, we identified the regions with a read coverage of 20× or higher in all exome-sequenced samples and termed them All-Well Covered Regions (All-WCR). The All-WCR contained a total of 16.76 Mb of exons and exon–intron boundaries. Only point mutations in these regions were taken into account when comparing sporadic and NF1-related tumors. Thirty somatic point mutations were identified in MPNST-NF1-001 and 15 in MPNST-NF1-002. However, the number of somatic point mutations was an order of magnitude higher in sporadic MPNST: 308 in MPNST-SP-001 and 257 in MPNST-SP-002 (Supplementary Fig S5A). Consequently, the mutational frequency in common regions was 1.79 and 0.89 mutations per megabase for MPNST-NF1-001 and MPNST-NF1-002, respectively, and 18.38 and 15.33 mutations per megabase for MPNST-SP-001 and MPNST-SP-002, respectively.

Among the total number of somatic point mutations identified in the PRS-WCR, the frequency of different types of base changes also differed between sporadic and NF1-related tumors. While mutations in NF1-related tumors did not accumulate any particular base change, sporadic tumors were highly enriched in C>T mutations (Supplementary Fig S5B), which represented 79.07 and 85.98% of the somatic mutations in MPNST-SP-001 and MPNST-SP-002, respectively. Analysis of the mutation context of C>T mutations in the sporadic primary tumors MPNST-SP-001 and MPNST-SP-002 (Supplementary Fig S5C) revealed an enrichment in TpC (56.78 and 57.48%, respectively) and a lower but significant enrichment in CpC (30.32 and 34.17%, respectively).

### Expression analysis

**Figure 6:**
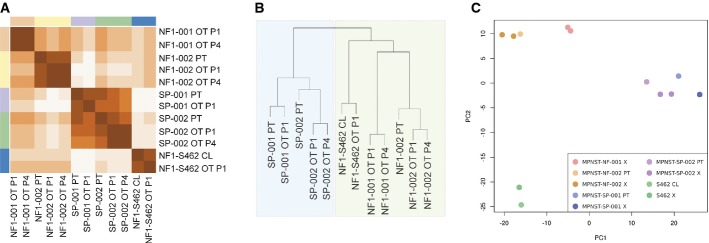
Gene expression profiles between primary and orthotopic xenograft MPNSTs are similar

Gene expression levels are influenced by different biological processes at the genomic and epigenomic levels. Thus, gene expression analysis can provide an integrative and more functional overview of the state of a tumor. Accordingly, expression array analysis was performed to validate the orthoxenograft models at the gene expression level. We observed a high global correlation between the normalized expression values for primary tumors and orthoxenografts at passage 1 (*R*^2^ ~0.9) and an even higher correlation between the values at passage 1 and passage 4 (*R*^2^ ~0.98), which is consistent with the removal of stromal cells (Fig6). We then analyzed a subset of ~1,000 genes representing a molecular signature associated with NF1-peripheral nerve sheath tumors, differentiating benign tumors from malignant tumors and derived cell lines (Miller *et al*, 2009). Using this signature, the analysis reported an even closer correlation between orthoxenografts and the corresponding primary tumors (*R*^2^ ~0.95); by contrast, using the same signature, lower correlation values were reported between distinct primary tumors (*R*^2^ ~0.78) and between primary tumors and non-corresponding xenografts (*R*^2^ ~0.8) (Fig6A). Unsupervised clustering analysis organized all of the samples analyzed, with each primary tumor grouped with the corresponding derived orthoxenografts at different passages (Fig6B). This analysis also classified the samples in two groups: NF1-related (including the S462 cell line and derived xenograft) and sporadic cases (Fig6B). This classification was obtained using both the molecular signature for NF1-related MPNST and the whole expression profile.

Heat map showing the correlations between expression levels of genes in the molecular signature of MPNSTs.

Hierarchical clustering of tumors and xenografts groups all primary tumors with their derived orthoxenografts. Moreover, sporadic tumors and NF1-related tumors form two different clusters.

PCA of genes in the molecular signature of MPNSTs. All primary-xenograft pairs cluster together. The first component distinguishes between sporadic and NF1-related tumors; the second component differentiates primary tumors (and derived orthoxenografts) from the cell line (and the derived orthoxenograft).

Finally, PCA was performed using the gene expression levels of the molecular signature; primary tumors were perfectly grouped with their corresponding derived orthoxenografts (Fig6C). At the same time, the first component separated NF1-associated MPNST and models from sporadic cases, while the second component separated primary tumors and orthoxenografts from the S462 cell line and its derived orthoxenograft (Fig6C).

### Using preclinical orthoxenograft MPNST models to test drug treatment regimens

**Figure 7:**
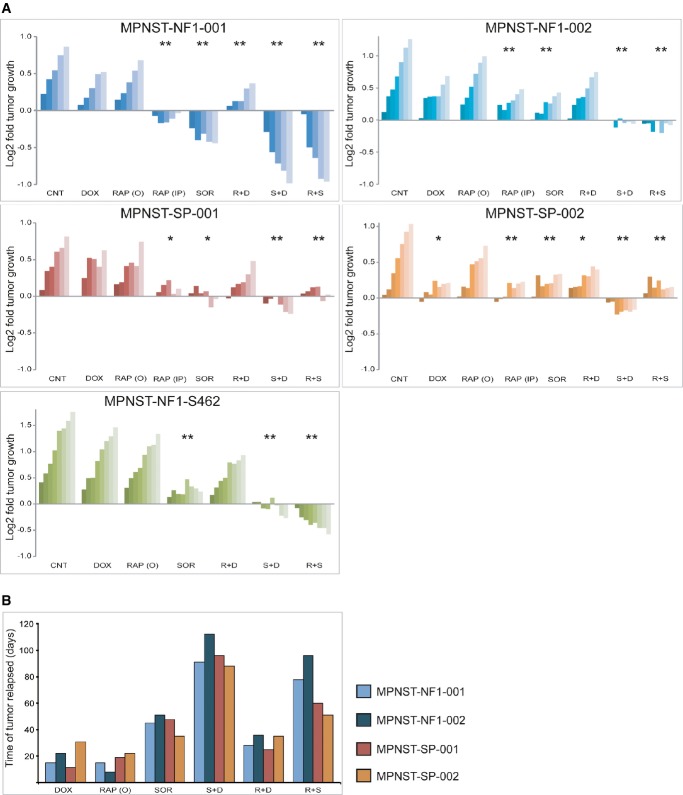
Preclinical orthoxenograft MPNST models to test drug treatment regimens

As the orthoxenografts tumor models were found to closely recapitulate the human disease at the histopathological, genomic, and transcriptomic levels, they were used to test clinically relevant therapeutic approaches. The four PDTX plus the orthoxenograft derived from the *NF1*-cell line S462 were treated in monotherapy with: (i) doxorubicin, the standard clinical chemotherapeutic agent, (ii) oral and (iii) intraperitoneal rapamycin, an allosteric mTOR inhibitor, and (iv) sorafenib, a BRAF inhibitor, or with combined drug regimens (v) doxorubicin + rapamycin, (vi) doxorubicin + sorafenib, and (vii) rapamycin + sorafenib. Both short- and long-term responses were evaluated (Fig7A and B, respectively).

Tumor growth effects of treatment with doxorubicin, sorafenib, rapamycin and combinations thereof in the five MPNST xenograft models. Results are plotted as an average of the log_2_ ratio of tumor volume at different days relative to the initial value. Statistically significant differences are shown as *P <* *0.05 and **P <* *0.001 versus control group by the Bonferroni test.

For long-term studies, a subgroup of treated mice (*n* = 3–5 mice/group) was kept alive for a maximum period of 4 months and sacrificed over time when relapsed tumor masses grew as large solid masses (usually 1,500–2,000 mm^3^). The graph illustrates differences in the time delay (in days) of relapsed tumor masses for the different treatments.

Doxorubicin as a single agent achieved a significant response relative to the placebo-treated animals in only one model (SP-002), whereas non-significant differences were observed in the other models. Intraperitoneal administration of rapamycin decreased tumor size in all tested models (we were unable to perform this drug treatment for the MPNST-NF1-S462 model due to a limited number of mice), whereas orally administrated rapamycin showed no effect on tumor growth. Sorafenib as a single therapy achieved a significantly better response than rapamycin and doxorubicin in all of the treated tumors. Notably, the combined sorafenib + doxorubicin and sorafenib + rapamycin treatments proved to be more active than either drug administered separately, suggesting a synergistic effect. Furthermore, the combined sorafenib + doxorubicin treatment achieved the most effective short-term response in all MPNSTs. Nevertheless, the sorafenib + rapamycin combination showed far more significant tumor reduction in *NF1*-related cases than in sporadic cases (Fig7A).

To investigate the long-term response, a subgroup of treated mice (*n* = 3–5 mice/group) were kept alive post-chemotherapy. Figure7B summarizes the time that tumor relapse took place for the four PDTX-MPNST orthoxenografts for each single and combined treatment. Long-term response studies confirm that combined sorafenib + doxorubicin treatment was the most effective treatment for the four models, suggesting a long-term synergistic antitumor response for combined therapy. Thus, while for sorafenib monotherapy treatments, tumor relapse took place in a period of 45–62 days, depending on the tumor (NF1-001, 45 days; NF1-002, 51 days; SP1-001, 48 days, and SP2-002, 35 days), for the combined sorafenib + doxorubicin treatment, tumor relapse ranged from 88 to 112 days (NF1-001, 91 days; NF1-002, 112 days; SP1-001, 96 days, and SP2-002, 88 days). Long-term response confirmed also the effectiveness of combined sorafenib + rapamycin treatment for the four PDTX, reinforcing their therapeutic effect, particularly in *NF1*-derived models.

At the time of sacrifice, histopathological changes were assessed in post-chemotherapy residual tumor masses for the different treatments. Analysis was performed in the tumor as well as in the surrounding stromal tissue for all treatments. To evaluate tumor response, the levels of necrosis and the number of mitosis were assessed (Table3, Supplementary Fig S6). Viable cells were identified in the residual masses of all treatments, although increased levels of necrosis were also seen for several treatments. The highest levels of necrosis were observed in sorafenib + doxorubicin and sorafenib + rapamycin treatments. Similarly, anti-proliferative effect was confirmed for all the combined treatments: a significant decrease was observed in the number of mitoses for the combined sorafenib + doxorubicin and sorafenib + rapamycin treatments relative to single drug treatments (Table3). Additionally, unlike PDTX orthoxenografts, the cell line-derived tumor showed highly mitotic index (Table3). Together, our results showed the efficacy of combined regimens using standard chemotherapy (doxorubicin) with target therapy agents (mainly sorafenib, but also rapamycin) in the treatment of the MPNST models described here.

**Table 3:** Characterization of histopathological response in post-chemotherapy tumor masses of MPNST tumors^a^

	MPNST-NF1-001		MPNST-NF1-002		MPNST-SP-001		MPNST-SP-002		MPNST-NF1-S462	
	Percentage of necrosis	No. of mitosis	Percentage of necrosis	No. of mitosis	Percentage of necrosis	No. of mitosis	Percentage of necrosis	No. of mitosis	Percentage of necrosis	No. of mitosis
Vehicle	0	8	0	12	18	26	7	6	10	99
Doxorubicin	5	5	0	3	20	19	0	2	10	27
Sorafenib	3	4	0	6	75	3	70	2	17	11
Rapamycin^b^	20	4	0	6	10	10	5	2	10	44
Doxorubicin + Sorafenib	35	3	25	2	85	5	74	0.6	24	7
Doxorubicin + Rapamycin^b^	30	9	0	7	30	14	10	2	12	12
Sorafenib + Rapamycin^b^	35	2	30	0.8	70	3	80	0.6	40	10

a For short-term drug response studies, the percentage of necrosis and the number of mitosis were evaluated by H&E staining of representative sections of the residual tumor masses of 3–5 mice per group, as an indicator of chemotherapeutic response. Four non-overlapping representative fields were counted per tumor.

b Oral administration of rapamycin.

Overexpression of *ABCB5* and *ASNS* has been linked to resistance to doxorubicin in different tumor types (Frank *et al*, 2005; Cheung *et al*, 2011). Exome-sequencing analysis revealed three putative mutations in the *ABCB5* gene (one non-sense and two missense variants) and one in the *ASNS* gene (a splice site variant) (Supplementary Table S2). The two variants with the clearest loss-of-function effect (the non-sense mutation in *ABCB5* and the splice site mutation in *ASNS*) were found in the same sporadic tumor (MPNST-SP-002). This tumor exhibited the best response to doxorubicin treatment, being potentially interesting for future pharmacogenetic studies.

### Cell line versus primary tumor orthoxenograft models

**Figure 8:**
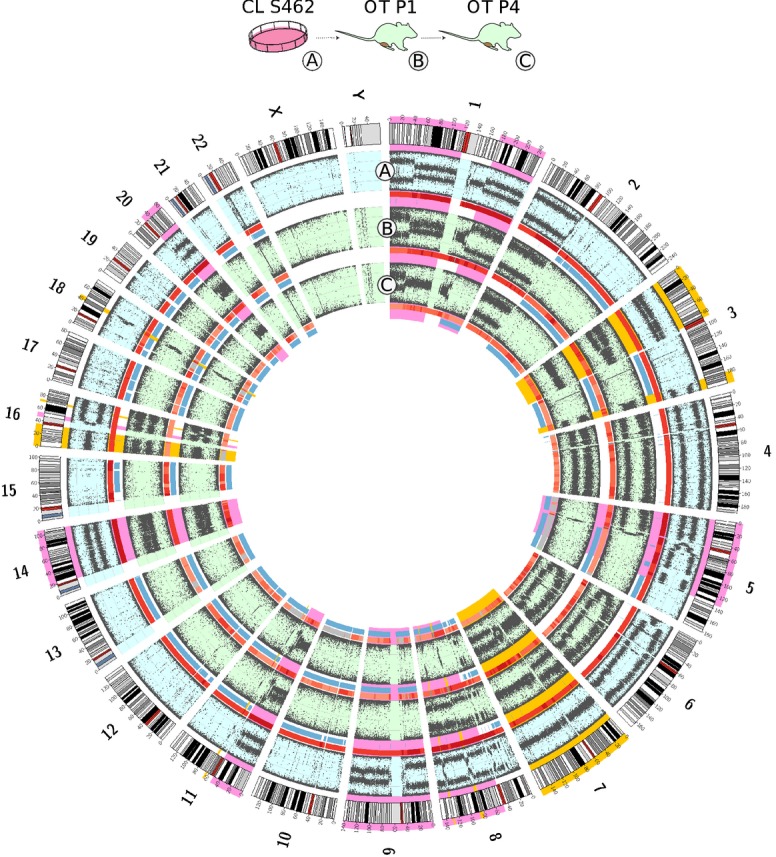
SNP array analysis of cell line S462 and its orthotopic xenograft tumors Orthotopic xenograft MPNSTs derived from cell line S462 showed a number of differences in genomic alterations when compared to the S462 cell line itself. The outermost layer shows the full set of canonical human chromosomes. The next layers, from outside to inside, show the BAF of the S462 cell line (A), and its derived xenograft at passages 1 (B) and 4 (C). Copy number variations are represented by a colored line under each BAF (gray: 2n, red: > 2n (chromosomal gain); green: < 2n (chromosomal loss). LOH events are shown in blue. Pink highlights mark the differences between cell line and xenografts compatible with a selection process, while orange highlights mark the regions consistent with structural genomic changes due to engraftment process and passaging.

In addition to the orthoxenograft models generated from primary MPNSTs, we also developed an orthoxenograft model from an established MPNST cell line (S462), following similar experimental procedures used for the other models. Histological characterization of the generated orthoxenograft showed that it retained the immunocytochemical marker characteristics of the original cell line, as well as reproducing the histological patterns of the NF1-associated orthoxenografts (Table2 and Supplementary Fig S1). At the molecular level, the number of point mutations and the expression pattern indicated a high degree of similarity between the cell line and the derived orthoxenografts (Figs5 and 6). However, at the structural genomic levels, the number of differences between the S462 cell line and the orthoxenografts at passages 1 and 4 was greater than observed in the MPNST-derived models (Fig8 and Supplementary Fig S4). These differences were classified in two groups. The first group contained genomic changes identical to those identified in models generated from primary tumors, that is, differences between orthoxenograft passages 1 and 4, consistent with structural genomic changes due to the successive engraftments (highlighted in yellow in Fig8). The percentage of genome affected by these changes was low and similar to that observed in the other models. The second group of differences corresponded to progressive changes along passages that were consistent with a selection process. We had previously observed a high diversity in the chromosomal content of S462 cells in culture by cytogenetic karyotyping (data not shown), and these progressive changes from primary tumor to passage 1 and passage 4 pointed to a clonal selection process, reducing the heterogeneity of the original cell culture (highlighted in magenta in Fig8).

## Discussion

MPNSTs are aggressive malignancies associated with poor survival and for which no effective therapy is available. We considered that establishing preclinical models was a useful step in developing an experimental framework for more accurate, personalized testing of new therapeutic approaches. Our molecular understanding of cancer has been significantly expanded in recent years thanks to the development of large-scale cancer genome initiatives such as TCGA or ICGC (International Cancer Genome Consortium *et al*, 2010) aimed at identifying the genomic alterations that drive the oncogenic process. However, the development of novel therapeutic strategies is largely contingent on the availability of preclinical models capable of recapitulating the disease. Orthotopic PDTXs have proved to be excellent models for this purpose because they preserve the key influence of the tumor microenvironment, in contrast to *in vitro* cellular models or subcutaneous xenografts (Richmond & Su, 2008; Kopetz *et al*, 2012; Tentler *et al*, 2012).

Histological analysis revealed a striking degree of concordance in the histopathological and immunohistochemical patterns of the primary tumor–xenograft pairs. Human stroma was rapidly lost after MPNST engraftment and replaced by murine stroma, in agreement with other reports (Xu *et al*, 1999; Sanz *et al*, 2009; DeRose *et al*, 2011; Hylander *et al*, 2013), greatly facilitating the genomic structural characterization of tumors, which is particularly crucial in the case of primary tumors with large proportions of normal cells. Molecular analysis at the genomic and transcriptomic levels also revealed a high degree of similarity between primary MPNSTs and their corresponding orthoxenografts. Genomic characterization confirmed that MPNSTs bear highly altered genomes (Mechtersheimer *et al*, 1999; Kresse *et al*, 2008; Mantripragada *et al*, 2009): an average of 75.7% of the genome was found to exhibit copy number alterations, with a high proportion of gains of whole chromosomes or large chromosomal regions and complex chromosomal rearrangements compatible with an origin in a catastrophic event (Stephens *et al*, 2011; Baca *et al*, 2013; Zhang *et al*, 2013). This view was supported by the fact that the complex genome structures remained remarkably stable throughout the establishment of the orthoxenografts and along xenograft passages and did not reflect permanent genomic instability. In fact, on average, < 6.8% of the genome structure showed copy number alterations or allelic imbalance changes in primary tumor–xenograft pairs. Exome analysis also revealed little difference in coding region point mutations between primary tumors and paired orthoxenografts, with a mean of 10 mutations in xenografts that were not present in the primary tumor. We took an additional step in validating the orthoxenograft models by analyzing biological status at the level of gene expression. Transcriptomic analysis of ~1,000 genes representing a molecular signature associated with MPNSTs and cell lines relative to normal Schwann cells and benign neurofibromas (Miller *et al*, 2006, 2009) showed a high correlation between primary tumors and paired orthoxenografts, even after several xenograft passages. All primary tumor–orthoxenograft pairs clustered together in an unsupervised cluster analysis and in a PCA, demonstrating the validity of the models.

The generation of an orthoxenograft using the NF1-related S462 MPNST cell line revealed differences in the engraftment process between the direct grafting of primary tumors at the sciatic nerve and the injection of cultured cell lines in the same site. Although the orthoxenograft generated from the S462 cell line reproduced the histological patterns of the NF1-associated orthoxenografts, genomic analysis showed progressive changes along passages consistent with a cellular and genetic selection process of the high heterogeneity present in *in vitro* cell cultures; a process that was not observed in the engraftment of primary tumors.

It is generally assumed that sporadic and hereditary cancer sharing common inactivated pathways may be biologically similar, although discussion is ongoing. The limited number of models presented here is too low to draw any conclusion comparing these two groups, since the effect of other intrinsic differences, such as the mutation in the *NF1* gene, the different age of the patients at diagnosis, or the loss of linked genes on chromosome 17 during LOH events, cannot be ruled out. Both MPNST types carried highly altered and rearranged genomes, but while NF1-associated MPNSTs seemed to have a higher degree of LOH than sporadic MPNSTs, the latter contained a number of point mutations an order of magnitude higher. Comparing the mutation frequencies with those obtained across all cancer types (Lawrence *et al*, 2013; Watson *et al*, 2013), the two NF1-associated MPNSTs developed in the same patient (0.89–1.79 mutations per Mb) fall in the low range of somatic mutation frequency, whereas the two independent sporadic MPNSTs (15.33–18.38) are in the highest frequency ranges. The high variation in mutation frequency within MPNSTs is also common in many other cancer types (Baca *et al*, 2013) and probably reflects current limitations in the classification of biological tumor properties. When analyzing the mutation spectra to identify signatures of carcinogenesis mechanisms, we identified a strong bias in sporadic MPNSTs toward a high frequency of C>T base substitutions, which is consistent with the action of APOBEC3 (Stenglein *et al*, 2010; Nik-Zainal *et al*, 2012; Taylor *et al*, 2013). The limited sequence data did not allow us to properly evaluate mutation clusters, so we do not know whether kataegis are present in the sporadic MPNST samples. Immunohistochemical characterization and gene expression analysis also revealed differences between the two patient-derived NF1 tumors and the NF1-associated MPNST cell line and the two independent sporadic MPNSTs. Particularly significant results were obtained from the unsupervised cluster analysis and the PCA using a molecular signature of ~1,000 genes associated with MPNSTs, which clearly separated the two MPNST types.

Finally, validated orthoxenograft MPNST models were used to test the effect of different drugs or drug combinations. The treatment experiments performed here demonstrated that the BRAF inhibitor sorafenib reduced MPNST growth. Sorafenib is clinically approved for the treatment of several cancer types such as kidney and liver cancer (Escudier *et al*, 2007; Llovet *et al*, 2008). At the preclinical level, good results have been reported in patients with advanced angiosarcomas or in mouse models of pancreatic islet cell tumors (Fendrich *et al*, 2012; Ray-Coquard *et al*, 2012). Sorafenib has been tested in MPNST cell lines *in vitro*, showing a significant inhibition of tumor growth, and data are available for *in vivo* models (Ambrosini *et al*, 2008). Altogether, these results strongly support the clinical evaluation of sorafenib in this subset of patients. A recent phase II clinical trial assessed the monotherapy activity of sorafenib in patients with different types of sarcomas, including 12 patients with MPNSTs. No objective responses were observed in nine of these patients, although two experienced a certain grade of regression of metastatic disease. Three patients showed stable disease, suggesting the drug had only a small effect (Maki *et al*, 2009). Although the results of the clinical trial do not appear very promising, sorafenib should be considered in combination with other agents, particularly taking into account the preclinical model results presented in this study. The antitumor activity of rapamycin and its analogs has been demonstrated in several *in vitro* studies with MPNST cell lines (Johansson *et al*, 2008; Zou *et al*, 2009; Endo *et al*, 2013) and in some clinical trials (Chan, 2004). Our results show that intraperitoneal rapamycin practically stopped tumor growth in almost all orthoxenografts. These results are consistent with other studies using patient-derived subcutaneous tumor xenografts (Bhola *et al*, 2010). However, oral rapamycin had no effect on tumor progression, in contrast to previous trials using subcutaneous xenografts derived from cell lines (Johansson *et al*, 2008). These results may illustrate the importance of the MPNST implantation site (subcutaneous or orthotopic) or reflect poor drug delivery to tumors by oral administration. In a recent clinical trial of temsirolimus (Okuno *et al*, 2011), only 5% of sarcoma patients responded to treatment, and the only patient with a MPNSTs did not respond. Although these are the first in-human results of this treatment for sarcomas, the numbers are too small to rule out the role of mTOR inhibitors as therapeutic agents, in particular for MPNSTs. As our results indicate, a combination of rapamycin and drugs targeting other pathways may be beneficial for MPNST patients. Doxorubicin seemed to show a mild synergistic effect in combination with rapamycin and with sorafenib, although it has already been administered alone as a conventional chemotherapy regime in patients with MPNSTs with poor outcomes (Ferner & Gutmann, 2002; Casali *et al*, 2008). Interestingly, the MPNSTs that showed the best response to doxorubicin treatment contained loss-of-function mutations in both *ABCB5* and *ASNS* (MPNST-SP-002). The identification of mutations in these genes, which are involved in resistance to drug treatment, opens the possibility of combining inhibitors of these proteins with chemotherapeutic agents to improve drug response.

To summarize, we developed, validated at the histological and genomic levels, and used five orthotopic patient-derived MPNST xenografts, which were found to be an excellent resource for preclinical investigation into this devastating tumor type. Our work points to differences in the engraftment processes of primary tumors compared with the engraftment of established cell lines. Results presented here evidenced biological differences between the two independent sporadic MPNSTs and the two NF1 MPNSTs developed in the same patient, in terms of genomic composition, mutation frequency and mutational signatures, immunohistochemical characterization, and gene expression, although the number of tumors and models analyzed here is still too low to drive any conclusion. Studies providing a comprehensive characterization of a larger number of sporadic and NF1-related MPNSTs are necessary to uncover possible differences between these two groups of MPNSTs that could eventually be translated into different therapeutic strategies. The most effective treatment tested in these preclinical models was sorafenib in combination with doxorubicin or rapamycin, which highlights the importance of combined drug therapy in achieving better therapeutic outcomes. Genomic characterization will enable us to use these orthoxenograft MPNST models in pharmacogenomic analysis.

## Materials and Methods

### Primary tumors and cell lines

Four fresh primary MPNSTs from three different patients were identified and removed at the Sarcoma Clinical Unit (UFTOS) of Bellvitge Hospital (HUB) and the Catalan Institute of Oncology (ICO), both institutions located on the IDIBELL campus. Two independent MPNSTs were from one NF1 patient, and the other two were from two different sporadic patients. After surgery, the tumor was sent to our pathology service where it was analyzed following standard protocols. Simultaneously, a piece of each tumor was stored in DMEM, 10% FBS culture medium at room temperature before being sent to our molecular unit. Once in our laboratory the tumor was divided into sections, processed, and preserved in order to have material for different purposes. Small pieces of each tumor were directly frozen in liquid nitrogen so that DNA, RNA, and/or protein could be obtained when needed. Small pieces were frozen in appropriate culture media so that cell culture experiments or mice engraftments could be performed. Informed consent was obtained from all subjects, and the study received the approval of the IDIBELL Ethics Committee.

### Animals

Six-week-old male nude Harlan mice weighing 18–22 g were used in this study. Animals were housed in a sterile environment, in cages with autoclaved bedding, food, and water. The mice were maintained on a daily 12-h light, 12-h dark cycle. All experiments with mice were approved by the IDIBELL Animal Care and Use Committee.

### Human MPNST implantation and perpetuation

Fresh surgical specimens from 4 human MPNSTs were implanted in athymic mice (Fig1). The donor tumors were minced into small fragments of 2–3 mm^3^ in size, and only macroscopically viable tumor tissue was implanted in the upper thigh (orthotopic implantation, OT). Under isoflurane anesthesia, a subcutaneous pocket was made with surgical scissors. Then, a small incision was made in the muscle to display the sciatic nerve. A piece of tumor was grafted there and grown surrounding the epineurium. The key points are that the tumor is fixed to the surface of sciatic nerve with synthetic monofilament, non-absorbable polypropylene suture (Prolene 7.0), and that the epineurium was not breached. Primary tumors were grafted orthotopically and subcutaneously in a minimum of three different mice in the first passage. After implantation, tumor formation was checked weekly by palpation. Depending upon the intrinsic characteristics of the primary tumor or cell line, orthotopic tumors became apparent 1–3 months after engraftment. Once orthotopic tumors had reached a volume of 1,000–1,500 mm^3^, mice were sacrificed and tumors were passed to another animal. Between seven and ten orthotopic mouse-to-mouse passages were performed for each orthoxenograft. For each passage, at least three mice were implanted in order to obtain a sufficient quantity of tumor material. After each passage tumors were frozen, paraffin-embedded, and cryopreserved to provide a source of viable tissue for future experiments.

The MPNST orthoxenograft procedure was approved by the campus Animal Ethics Committee and complied with AAALAC (Association for Assessment and Accreditation of Laboratory Animal Care International) procedures.

The S462 cell line was kindly provided by Dr. Nancy Ratner. S462 belongs to a NF1 patient carrying the c.6792C>A non-sense mutation in exon 37. This patient developed a grade IV MPNST on the thigh at age 19. The MPNSTs carried LOH in the *NF1*, *TP53*, and *CDK2NA* genes. Cell line establishment was previously described (Frahm *et al*, 2004). To establish the orthoxenograft model from the S462 NF1-MPNST cell line, we injected 0.3 ml of the cell suspension (3 × 10^6^ cells) with a needle directly in the upper thigh muscle using Matrigel (a solubilized tissue basement membrane matrix rich in extracellular matrix proteins). This enabled tumor growth around the epineurium.

A total of 17 samples were obtained and analyzed in different experiments: four primary MPNSTs, one cell line, six orthoxenografts in passage 1 and 6 orthoxenografts in passage 4 (for one of the models, MPNST-NF1-001, two independent engraftments at passages 1 and 4 were analyzed). Details of all tumors and the cell line are provided in Table1.

### Presence of metastases

To investigate the capability of the orthoxenograft tumors to disseminate in mice, the lungs, livers, and brains of 45 animals were histologically examined by H&E staining. The five models were examined after sacrifice for the presence of synchronic micrometastases.

A subgroup of 15 orthotopically implanted mice (the two sporadic models and MPNST-NF1-001) was kept alive for 4–6 months after tumor removal to investigate the dissemination capabilities over a longer time frame (metachronic micrometastases).

### Nucleic acid preparation

#### DNA

GentraPuragene Kit (Qiagen) was used for DNA isolation of frozen human and xenograft tumors, according to manufacturer's recommendations, after homogenization using TissueLyser (Qiagen). DNA quality and quantity were assessed by visual inspection in an agarose gel and with NanoDrop and PicoGreen.

#### RNA

Total RNA was isolated from frozen samples using miRCURY RNA (Exiqon). RNA integrity number (RIN) was verified for each sample using a RNA Nano Chip Kit (Agilent Technologies, Germany) in Agilent Bioanalyzer 2100.

### Immunohistochemistry analysis

Paraffin-embedded human primary and mouse orthoxenograft MPNST sections (3–5 μm) were deparaffinized in xylene and gradually rehydrated. Endogenous peroxidases were blocked by incubation with hydrogen peroxide (H_2_O_2_ 3%, for 20 min), and antigen retrieval was performed by heating tissue sections for 20 min in citrate buffer (pH = 6 or pH = 9 depending on the antibody manufacture's protocol). Blocking was performed by incubation for 20 min with 10% horse serum. The primary antibodies vimentin (1:500, IR630, DAKO), Desmin (IR606, DAKO), actin (1:50, M0851, DAKO), EMA (1:200, IR629, DAKO), CD34 (IR632, DAKO), S100 (IS504, DAKO), P53 (IR616, DAKO), and Ki-67 (1:75; M7240, DAKO) were incubated overnight at 4°C following the manufacturer's guidelines. Secondary HPRT-conjugated antibody (Envision, DAKO, Denmark) was incubated at room temperature for 30 min. Finally, development was performed by incubation with diaminobenzidine (DAB) (DAKO, Denmark) for 10 min. Nuclei were counterstained with hematoxylin. For stroma analysis, primary antibodies rat anti-mouse CD34 (1:100, 8158, Abcam) and mouse anti-human CD34 (1:100, 8536, Abcam) were incubated overnight at 4°C. Secondary HPRT anti-mouse-conjugated antibody (Envision, DAKO, Denmark) or biotinylated anti-rat (Daki, Denmark; 1:200 dilution) were incubated at room temperature for 60 min.

### Sanger sequencing

c.350T>A mutation region of blood, primary tumors, and orthotopic xenograft tumor was sequenced by PCR amplification using specific primers targeting the mutation region of the *NF1* gene and the BigDye Terminator v.3.1 Sequencing Kit (Applied Biosystems, Carlsbad, CA). Sequences were analyzed on an ABI Prism 3100 Genetic Analyzer (Applied Biosystems, CA, USA).

### SNP array analysis

SNP array analysis was performed on all 17 samples using Beadchip technology from Illumina, but with different chips depending on availability at the time of the analysis (Supplementary Table S3). In particular, two samples were analyzed using Illumina Human660W-Quad chip (655,246 SNPs), six samples using Illumina HumanOmniExpress v1 (730,525 SNPs), and nine samples using Illumina HumanOmni1S (1,185,076 SNPs). In all cases, raw data were processed with Illumina Genome Studio v2009 with the Genotyping module v1.1.9 to extract B-allele frequency (BAF) and log R ratio (LRR) values for each SNP.

SNP array data were analyzed using the R package ASCAT (Van Loo *et al*, 2010) to obtain loss-of-heterozygosity (LOH) and allele-specific copy number (CN) profiles from the BAF and LRR values. All samples were analyzed independently and treated as unpaired samples, using the germline genotype prediction functionality from ASCAT. In short, after loading BAF and LRR data, the germline genotype parameters were estimated and the data were segmented using the ASPCF algorithm. Next, ASCAT computed the most likely combination of CN states, total ploidy and percentage of aberrant cells. Circular genomic plots were created using Circos (Krzywinski *et al*, 2009).

### Exome sequencing

Exome sequence capture and amplification was performed in all primary tumors and constitutional DNA from the different patients, in the S462 cell line, and in all orthoxenograft models at passage 1 and NF1-MPNST-001 passage 4, using Agilent SureSelect Human All Exon kit (Agilent, Santa Clara, CA, US) according to the manufacturer's instructions. Paired-end sequencing was performed on a HiSeq2000 instrument (Illumina) using 76-base reads. Reads were aligned to the reference genome (GRCh37), and a BAM file was generated using SAMtools. PCR duplicates were removed using SAMtools and custom scripts, and single-nucleotide variant calling was performed using a combination of SAMtools and Sidrón as described previously (Puente *et al*, 2011). For orthoxenograft-derived samples, reads were first aligned to mouse genome (mm9), and those read-pairs which did not align to mouse were then aligned to the human genome following the same pipeline as above. This procedure removed murine-derived reads, which might interfere in the analysis by artificially increasing the number of variants. However, this could lead to the removal of certain human genes with a very high DNA sequence identity to mouse DNA and caused some true changes to be overlooked. Variants detected in the tumor sample that were not present in the matching constitutional DNA were considered somatic variants (Supplementary Table S1). For the validation analysis, only mismatch variants were taken into account. Common variants, defined as those present in dbSNP135 with a minor allele frequency > 1%, were filtered out. For all variants identified in primary tumors and in the orthoxenografts, bam-readcount was used to check whether they were supported by a read in the other related samples; variants were considered to be present if there was at least one read with a quality of over 20. These data were used to identify somatic mutations as well as gained mutations (variants present in the xenograft but not found in the primary tumor) and lost mutations (variants identified in the primary tumor but not detected in the xenograft). Analysis of the genomic context of the C>T variants was performed using R and Bioconductor.

### Expression microarray analysis

Gene expression profiles were determined using Affymetrix Human Gene 1.0 ST arrays (Affymetrix, Santa Clara, CA) following standard protocols. Expression data were analyzed using R version 3.0.2 (Dean & Nielsen, 2007) and the Bioconductor (Gentleman *et al*, 2004) package Affy (Gautier *et al*, 2004). Raw CEL files were normalized with RMA, and the normalized expression values were extracted. Samples were compared at the level of normalized expression values using the Pearson correlation coefficient to quantify the changes between primary tumors and the derived orthoxenografts. Expression profiles were classified using a hierarchical clustering approach with Euclidean distance and average as agglomeration method. Heatmaps represent the Pearson correlation between pairs of samples and were drawn using the gplots library.

### Drug treatment

To prepare each drug treatment, an early-passage (P2–P4) orthoxenograft tumor had to be expanded. To do this, each tumor was implanted in five mice. When tumors reached a minimum size of 1,000–1,500 mm^3^, mice were sacrificed, tumors were harvested and cut into small fragments, and the tumor fragments were grafted into 50–70 mice, depending on the size of the experiment. When the tumors reached a homogeneous size of 1,000–1,500 mm^3^, they were randomly distributed into different treatment groups (*n* = 7–10/group). Seven treatment regimens were tested: (i) doxorubicin; (ii) intraperitoneal rapamycin; (iii) oral rapamycin; (iv) sorafenib; (v) doxorubicin plus oral rapamycin; (vi) doxorubicin plus sorafenib; and (vii) oral rapamycin plus sorafenib. Drugs were administered as follows: mice were given an intraperitoneal injection of doxorubicin (8 mg/kg) once, at the beginning of the treatment; a daily oral or intraperitoneal dose of rapamycin (5 mg/kg); and a daily oral gavage dose of sorafenib (60 mg/kg). Rapamycin was obtained from Novartis Pharmaceuticals Corporation (East Hanover, NJ). Rapamycin was administered in a microemulsion solvent composed of 50% olive oil for the oral dosage and diluted in 10% DMSO; for the intraperitoneal dosage, it was diluted in 0.5% w/v carboxyl methylcellulose (Sigma; Johansson *et al*, 2008). Sorafenib was purchased from LC Laboratories (Woburn, MA) and dissolved in 50% cremophor EL (Sigma, St. Louis, MO)—50% ethanol. The mixture was vortexed for 30–60 min to dissolve sorafenib and then dissolved in 75% water immediately prior to oral gavage (Wu *et al*, 2012). Doxorubicin dose was chosen on the basis of studies in which intraperitoneal administration at 8 mg/kg was tested in a xenograft model derived from a MPNST cell line (Johansson *et al*, 2008). Rapamycin administered intraperitoneally at 5 mg/kg was used previously in a genetically engineered MPNST mouse model (Johannessen *et al*, 2008), and the sorafenib dose was chosen on the basis of preclinical studies in which daily oral administration of Sorafenib at 30–60 mg/kg was tested in several tumor models (Wilhelm *et al*, 2004). A mouse control group receiving no drug was used for each drug treatment experiment. In the first drug experiment, using mice MPNST-NF1-001, two additional control groups were treated with the two vehicles for oral rapamycin and sorafenib administration. No significant differences in tumor response were observed between the different vehicles relative to the untreated group. Thus, to simplify the presentation of data, we have included only one control group per model, which corresponds to the untreated animals.

In the drug response and metastasis experiments, the majority of mice were sacrificed when tumors reached sizes between 2,000 and 2,500 mm^3^. After sacrifice, tumors were dissected out, measured, and weighed. Representative fragments were frozen in liquid nitrogen and fixed and paraffin-embedded.

Overall treatment time varied slightly between experiments, between 12 and 25 days, according to the intrinsic differences in tumor growth. The duration of the drug response treatment was always marked by the tumor size of the matching control group, which complied with our institution's and international standard animal ethics protocols. Briefly, MPNST-NF1-001 treatments lasted 12 days, MPNST-NF1-002 treatments lasted 22 days, MPNST-SP-001 treatments lasted 14 days, MPNST-SP-002 treatments lasted 25 days, and MPNST-NF1-S462 treatments lasted 19 days. After treatment initiation, tumors were measured using a caliper every 2–3 days and tumor volume was calculated using the formula *v* = (*w*^2^ l/2), where *l* is the longest diameter and *w* the width. Changes in tumor volume were quantified as the log_2_ ratio between observed and baseline volume. The rate of change in volume across different treatment categories was modeled using linear mixed models (LMM). The interaction between follow-up time (in days) and treatment was used to assess the effect of each treatment in terms of volume change and compared to the control group. Significance was tested by the Wald test, and *P*-values were adjusted by Bonferroni's correction to address the problem of multiple comparisons due to multiple testing (Supplementary Table S4). All tests were two-sided, and significance level was set at 0.05. Analyses were also repeated after exclusion of mice that died during follow-up, with no appreciable impact on results (data not shown). The analyses were performed using Stata v10 (StataCorp LP, College Station, Texas).

For long-term studies, a subgroup of treated mice (*n* = 3–5 mice/group) was kept alive for a maximum period of 4 months and sacrificed over time when relapsed tumor masses grew as large solid masses (usually 1,500–2,000 mm^3^). For the characterization of histopathological response in post-chemotherapy MPNSTs, we examined all cases to evaluate the following histological features: mitotic rate expressed as the number of mitotic figures per 10 high-power fields (1 field, 0.164 mm^2^) and necrosis. For each tumor, mitotic rate and necrosis were estimated on whole transverse sections from three areas (Dutour *et al*, 2009).

**The paper explained**

**Problem**

Malignant peripheral nerve sheath tumors (MPNSTs) are aggressive soft-tissue sarcomas with poor survival for which no effective therapy is available. In 50% of cases, they occur in the context of neurofibromatosis type I; the remainder arises sporadically. Current *in vivo* tumor models of MPNSTs are limited to models derived from established cancer cell lines. *In vivo* models are needed that better recapitulate human MPNSTs and that could be used to assess effective, standardized therapies.

**Results**

We generated five distinct MPNST orthoxenograft models (four are patient-derived tumor xenografts (PDTX) and one is an orthoxenograft derived from an NF1-related MPNSTs cell line) that were exhaustively characterized by histopathological analysis, SNP array, exome sequencing, and expression array analysis. We demonstrated that all orthoxenograft models recapitulate each of the features of their parental primary tumors and proved that they are excellent preclinical models for drug treatment trials. Finally, therapeutic experimentation with sorafenib—either alone or, more effectively, in combination with doxorubicin or rapamycin—greatly reduced tumor growth in all models, supporting their use to treat patients with MPNSTs.

**Impact**

Our work reports the creation of the first patient-derived MPNST orthoxenograft model resource available for preclinical testing. The results strongly support the clinical use of sorafenib in these patients.

## Supporting Information

Supplementary Table S1

Review Process File
