# Supplementary material for: Comprehensive establishment and characterization of orthoxenograft mouse models of malignant peripheral nerve sheath tumors for personalized medicine
Source: EMBO Mol Med. 2015 Mar 25;7(5):608–27. doi: 10.15252/emmm.201404430 (PMC4492820; doi:10.15252/emmm.201404430)
Supplement: Supplementary file 1 [file emmm0007-0608-sd1.pdf]

Supplemental Information:

Table of Contents

Supplementary Figure Legends.. 3

Figure S1: Orthoxenograft mouse MPNST models closely resemble primary tumor...... 3

Figure S2: Distal dissemination of MPNST tumors orthotopically engrafted in nude mice ………………………………………………………………………………………... 3

Figure S3: Human stroma is lost after engraftment and replaced by murine cells.. 4

Figure S4: Orthotopic xenograft MPNST maintain genomic alterations found in primary tumors.. 4

Figure S5: Mutational signatures of primary tumors... 5

Figure S6: Histopathological characterization of residual tumor masses post-chemotherapy... 5

Supplementary Figures and Tables... 6

Figure S1.. 6

Figure S2.. 7

Figure S3.. 8

Figure S4A.. 9

Figure S4B.. 10

Figure S5 11

Figure S6 12

Table S1 13

Table S2 13

Table S3 14

Table S4 15

Figure S1. **Orthoxenograft mouse MPNST models closely resemble primary tumors**

A. Orthotopic MPNST xenografts at passages 1 (OT P1) and 4 (OT P4) are histopathologically similar to their corresponding primary MPNST (PT) in hematoxylin-eosin staining of paraffin sections. Main panels show a general view of tumors at low magnification (40x), inset pictures were taken at higher magnification (400x).

B. Orthotopic xenograft and primary MPNST exhibit similar immunohistochemical features. A representative immunostained section of vimentin, CD34, S100 and p53 is shown from primary tumors (PT), cell line S462 (CL) and orthotopic tumors (OT) corresponding to patients MPNST-NF1-001, MPNST-SP-001 and MPNST-NF1-S462. Positive antibody signals are shown in brown, and the hematoxylin counterstain in blue. Main panels show pictures at high magnification (400x) and inset pictures display mitotic cells present in the tumors.

Figure S2. Distal dissemination of MPNST tumors orthotopically engrafted in nude mice

Orthoxenografts of MPNST reproduce distal dissemination properties of primary MPNST tumors. **A.** Synchronous micrometastases in the lung were identified after mouse sacrifice in MPNST-SP-001, MPNST-SP-002 and the cell-line derived xenograft MPNST-NF1-S462. Representative hematoxylin-eosin staining of paraffin sections from orthoxenograft distal dissemination tumors at low (x100) and high (400x) magnifications. To better characterize the metastasic phenotype, one of the synchronous micrometastases of MPNST-SP-002 was immunochemically characterized using four antibodies, vimentin, CD34, S100 and Ki-67, which are a perfect match with the corresponding orthoxenograft MPNST (upper-right panels).

B. Metachronous micrometastasis in the lung was identified after mouse sacrifice in a mouse kept alive for 4-6 months after tumor removal in the MPNST-SP-002 model. Panel shows a representative hematoxylin-eosin staining of paraffin sections from orthoxenograft distal dissemination tumor at low (x100) and high (400x) magnifications.

Figure S3. Human stroma is lost after engraftment and replaced by murine cells

Stromal elements of the primary tumors are labeled with anti-human CD34 but not with anti-mouse CD34; patient-derived xenograft is labeled with anti-mouse CD34 only and no anti-human marker. Two representative sections (at 40x and 400x magnification) of the primary tumors (PT) and the orthoxenograft tumors at passages 1 (OT P1) and 4 (OT P4) were labeled with both anti-human CD34 (H) and anti-mouse CD34 (M).

Figure S4. Orthotopic xenograft MPNST maintain genomic alterations found in primary tumors

Genome-wide SNP array profiles of **A.** MPNST-NF1-001 and MPNST-NF1-002 and **B**. MPNST-SP-001 and MPNST-SP-002 are shown as Circos plots. The outer layer shows the full set of autosomal human chromosomes; the following layers, from outside to inside, show the allele frequency of the primary tumor (A), and the derived xenografts at passages 1 (B) and 4 (C). Copy number variations are represented by a colored line under each allele frequency (Grey = 2n, Red = >2n (chromosomal gain); Green = <2n (chromosomal loss). LOH events are shown in blue. Changes not conserved between primary and xenograft tumors are marked in orange.

Figure S5. Mutational signatures of primary tumors

A. Number of somatic variants in All-WCR (regions over 20x in all samples) of the four primary MPNST.

B. Base substitution for all somatic mismatch variants in PRS-WCR (regions over 20x in all samples related to a primary tumor).

C. Genomic sequence context of all C>T substitutions in the PRS-WCR of the sporadic MPNSTs.

Figure S6. Histopathological characterization of residual tumor masses post-chemotherapy

Hematoxylin-eosin staining of paraffin sections from orthoxenograft treated tumors are shown as an indicator of chemotherapy response (percentage of necrosis and number of mitosis).

Left panels show a general view of tumors at low magnification (40x) and right panel shows pictures taken at higher magnification (400x) for MPNST-NF1-001, MPNST-NF1-002, MPNST-SP-001 and MPNST-SP-002 orthoxenografts treated with vehicle (CNT), doxorubicin (DOX), sorafenib (SOR), rapamycin (intraperitoneally administered) (RAP) and combined doxorubicin+sorafenib (D+S), doxorubicin+rapamycin (D+R) and rapamycin+sorafenib (R+S), respectively. Four non-overlapping representative fields were counted for each sample, 3–5 mice by treatment group were analyzed.

Supplementary Figure 1

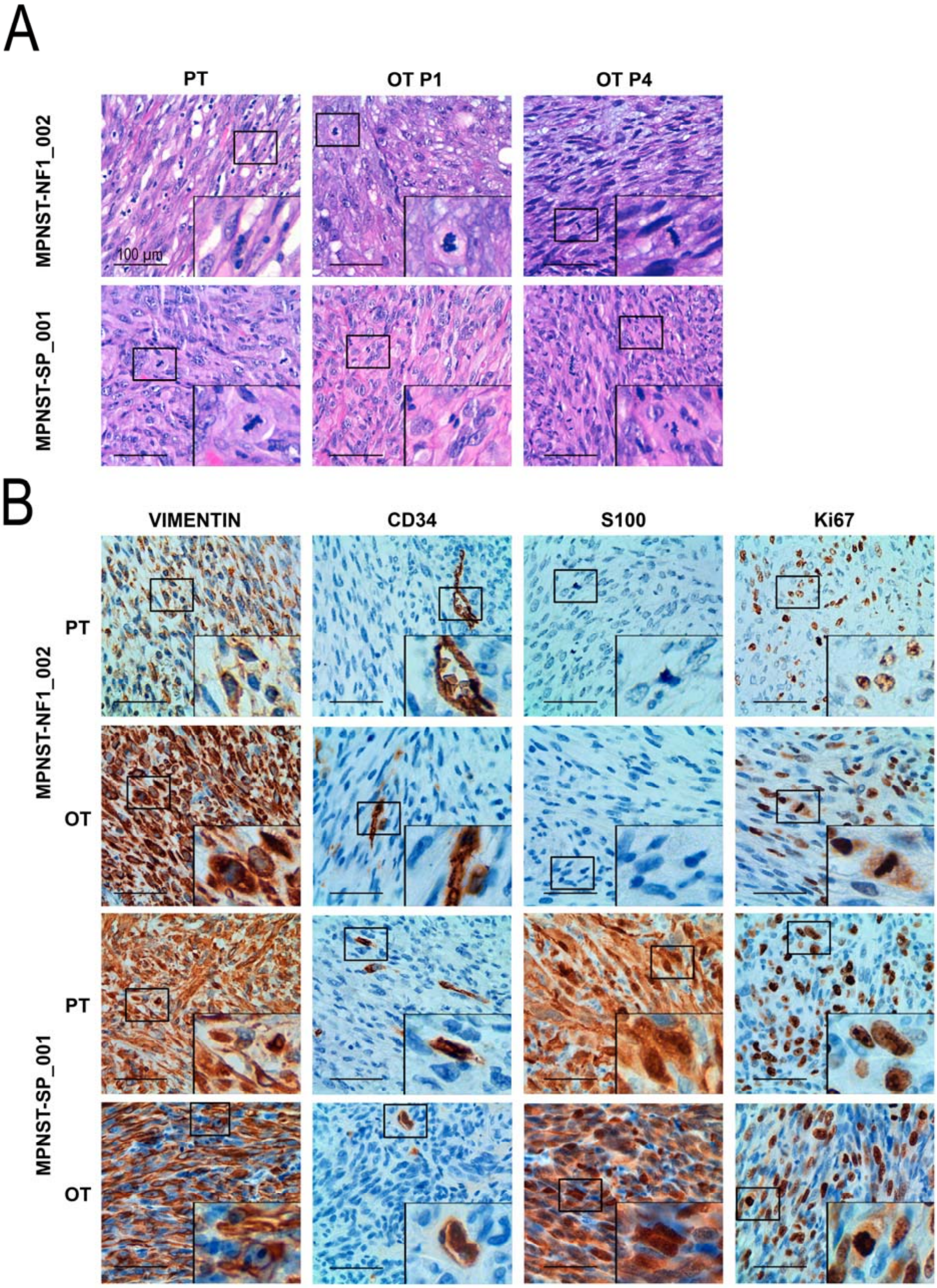

Supplementary Figure 2

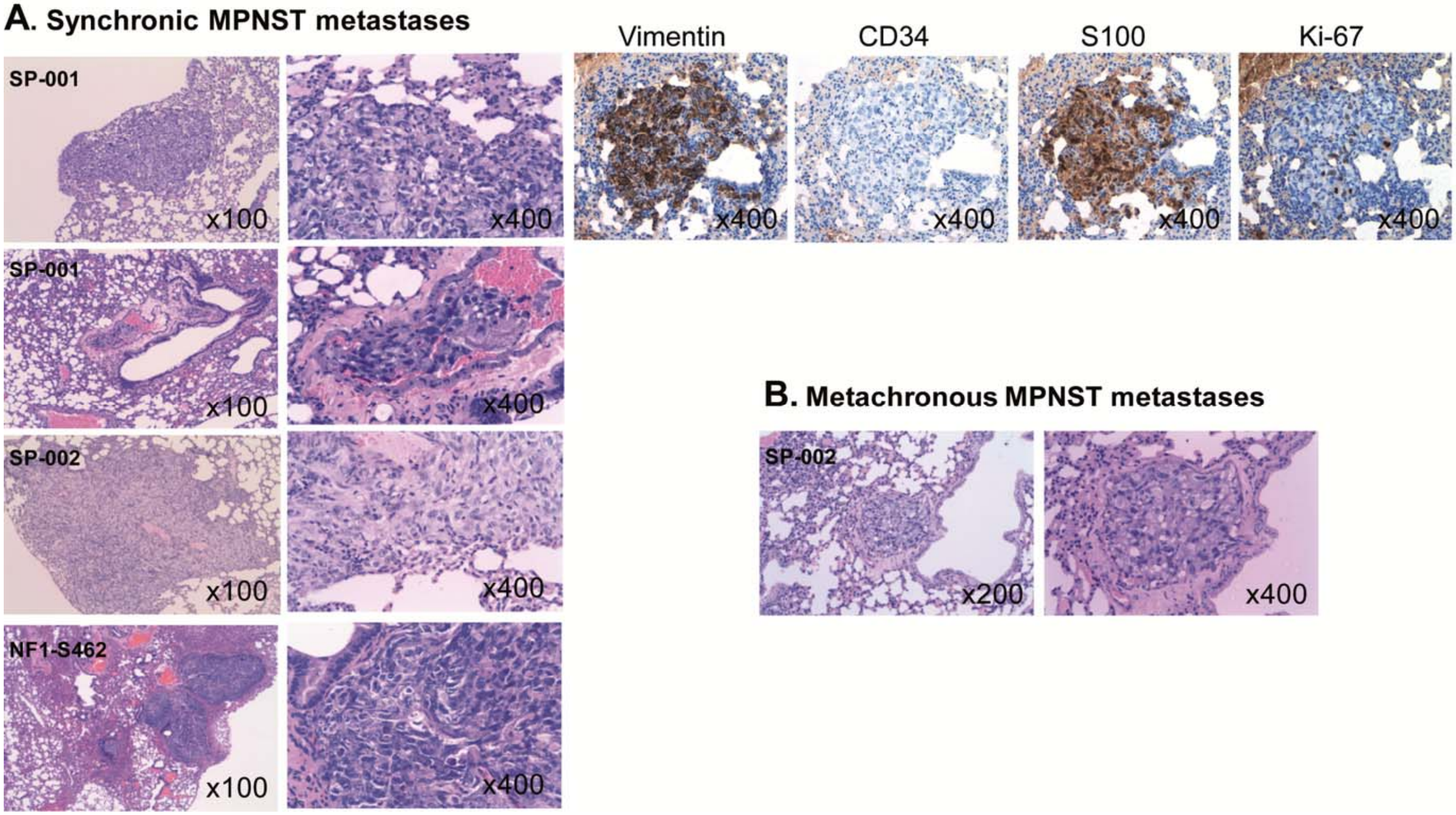

Supplementary Figure 3

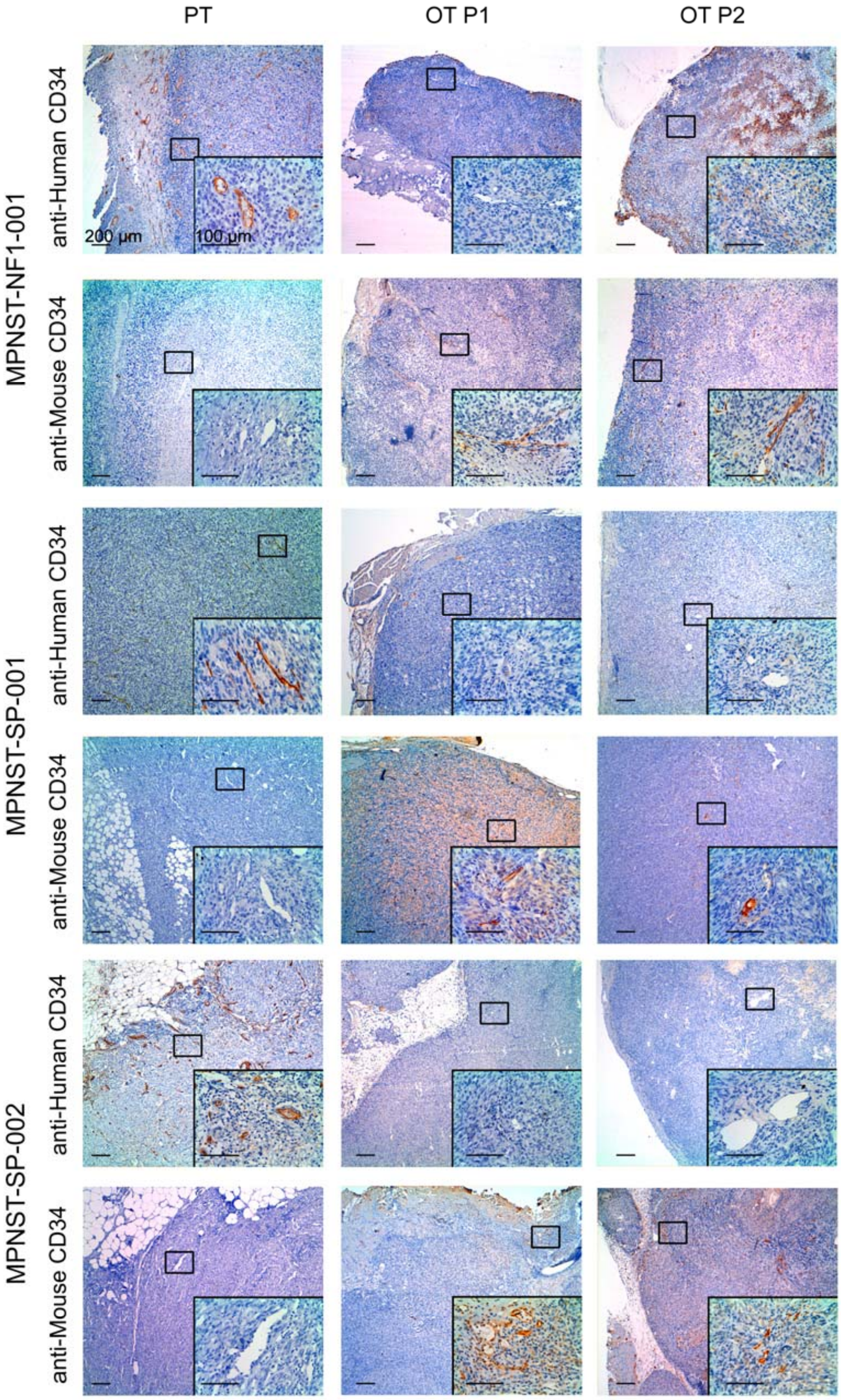

Supplementary Figure 4

A

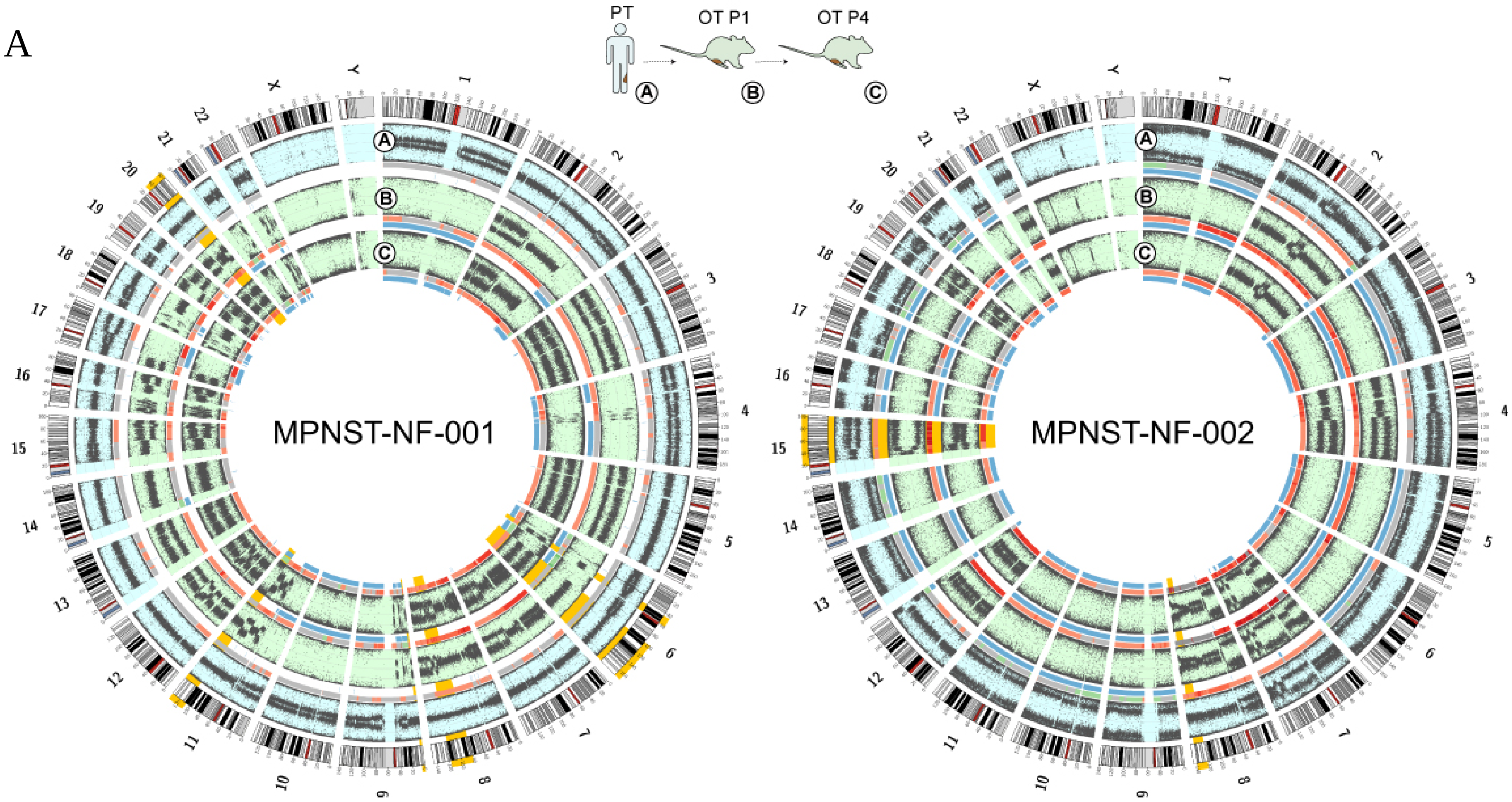

B

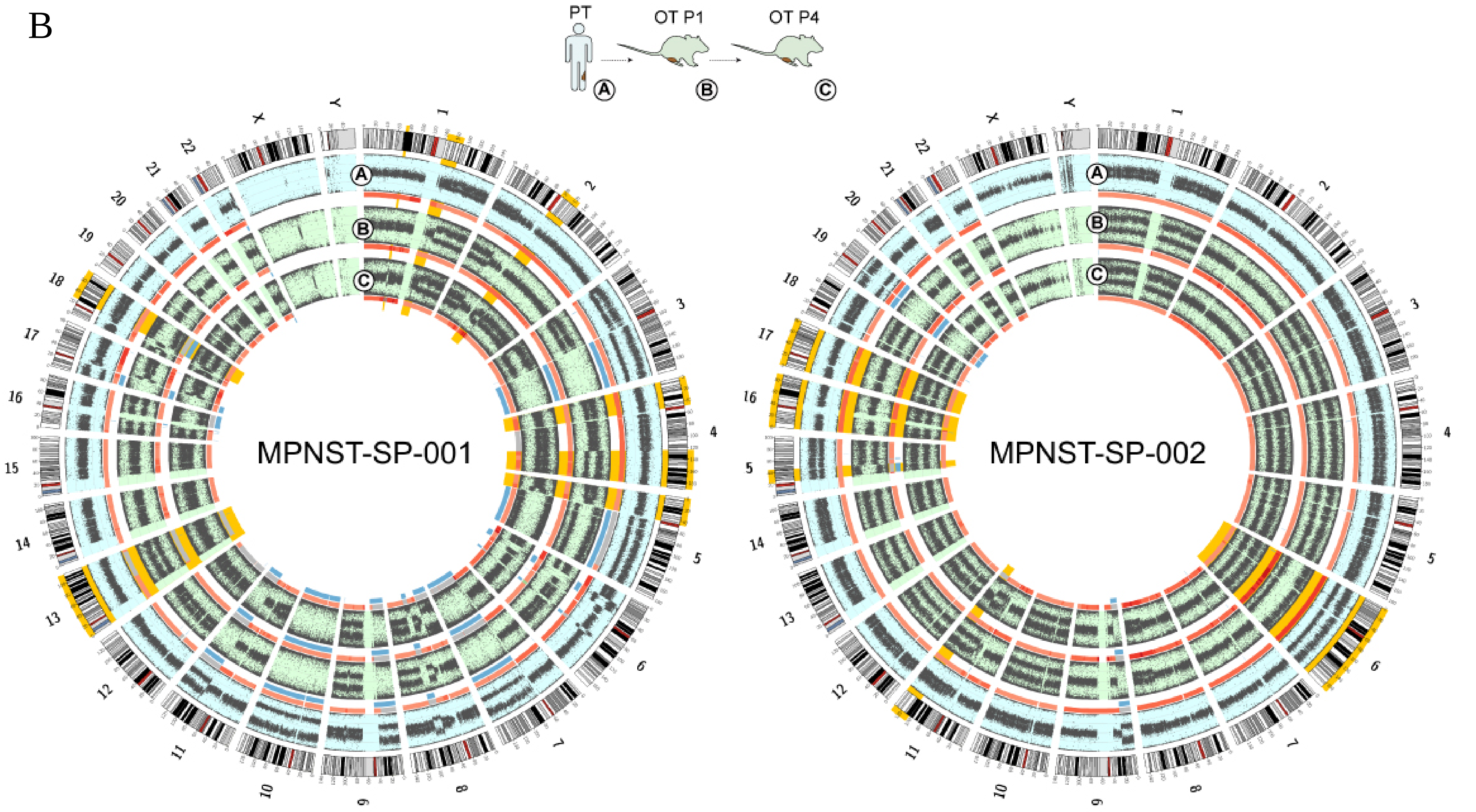

Supplementary Figure 5

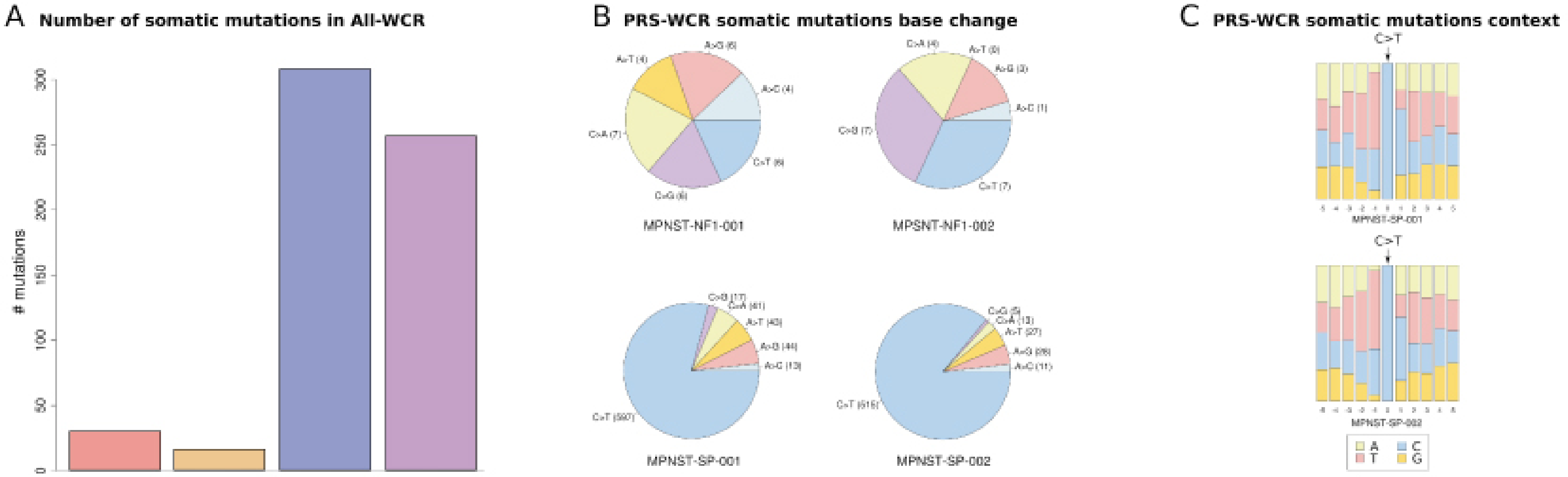

Supplementary Figure 6

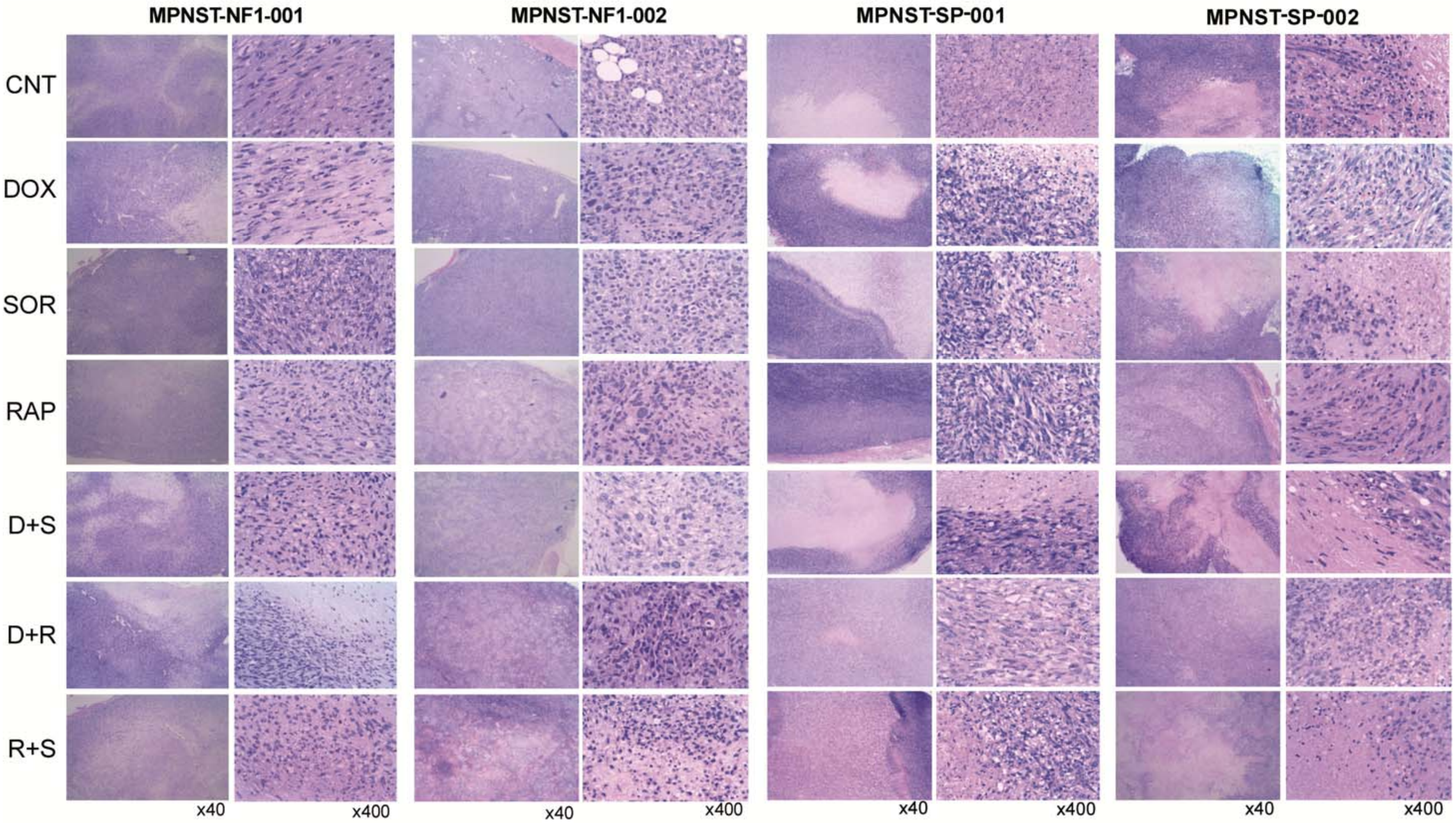

Table S1. Somatic mutations identified in PRS-WCR.

This table is presented in an Excel format.

Table S2. Mutations probably related to doxorubicin resistance.

**Gene**	**Mutation DNA level**	**Mutation protein level**	**Tumor sample**
ABCB5	c.43C>T	p.Gln15*	MPNST-SP-002
ABCB5	c.37A>T	p.Asn13Tyr	MPNST-SP-002
ABCB5	c.643C>T	p.Pro215Ser	MPNST-SP-001
ASNS	c.611-1G>A	-	MPNST-SP-002

Table S3. BeadChip versions used for the SNP array analysis of 17 samples.

PATIENT	TUMOR	SNP ARRAY CHIP
MPNST-NF1-001	TP	Human660W-Quad BeadChip
	OT 1A	Illumina OmniExpress
	OT 1B	Illumina OmniExpress
	OT 4A	Illumina OmniExpress
	OT 4B	Illumina OmniExpress
MPNST-NF1-002	TP	Illumina Omni1S
	OT 1	Illumina Omni1S
	OT 4	Illumina Omni1S
MPNST-SP-001	TP	Illumina OmniExpress
	OT 1	Illumina Omni1S
	OT 4	Illumina Omni1S
MPNST-SP-002	TP	Illumina OmniExpress
	OT 1	Illumina Omni1S
	OT 4	Illumina Omni1S
MPNST-NF1-S462	CL	Human660W-Quad BeadChip
	OT 1	Illumina Omni1S
	OT 4	Illumina Omni1S

Table S4. P-values of the different drug treatments using the Wald test (adjusted by Bonferroni correction).

	MPNST-NF1-001		MPNST-NF1-002		MPNST-SP-001		MPNST-SP-002		MPNST-NF1-S462	
	n	p-value	n	p-value	n	p-value	n	p-value	n	p-value
**CNT**	14	NS	7	NS	5	NS	5	NS	5	NS
**DOX**	8	NS	5	NS	5	NS	3	0.02	7	NS
**RAP (O)**	9	NS	5	NS	6	NS	4	NS	7	NS
**RAP (IP)**	6	<0.0001	6	0.01	6	<0.0001	6	0.001		NS
**SOR**	9	<0.0001	6	0.03	9	<0.0001	9	<0.0001	7	<0.0001
**R + D**	10	<0.0001	5	NS	6	NS	5	0.003	9	NS
**S + D**	10	<0.0001	7	<0.0001	9	<0.0001	9	<0.0001	9	<0.0001
**R + S**	7	<0.0001	7	<0.0001	9	<0.0001	10	<0.0001	7	<0.0001
