# Supplementary material for: Comprehensive establishment and characterization of orthoxenograft mouse models of malignant peripheral nerve sheath tumors for personalized medicine
Source: EMBO Mol Med. 2015 Mar 25;7(5):608–27. doi: 10.15252/emmm.201404430 (PMC4492820; doi:10.15252/emmm.201404430)
Supplement: Supplementary file 3 [file emmm0007-0608-sd3.pdf]

EMBO Molecular Medicine

Comprehensive establishment and characterization of orthoxenograft mouse models of malignant peripheral nerve sheath tumors for personalized medicine

Joan Castellsagué, Bernat Gel, Juana Fernández-Rodríguez, Roger Llatjós, Ignacio Blanco, Yolanda Benavente, Diana Pérez-Sidelnikova, Javier García-del Muro, Joan Maria Viñals, August Vidal, Rafael Valdés-Mas, Ernest Terribas, Adriana López-Doriga, Miguel Angel Pujana, Gabriel Capellá, Xose S. Puente, Eduard Serra, Alberto Villanueva, Conxi Lázaro

Corresponding author: Conxi Lazaro, Catalan Institute of Oncology (ICO-IDIBELL)

Review timeline:

Submission date:	13 August 2014
Editorial Decision:	16 September 2015
Revision received:	04 January 2015
Editorial Decision:	06 February 2015
Revision received:	12 February 2015
Editorial Decision:	17 February 2015
Revision received:	24 February 2015
Accepted:	25 February 2015

Transaction Report:

(Note: With the exception of the correction of typographical or spelling errors that could be a source of ambiguity, letters and reports are not edited. The original formatting of letters and referee reports may not be reflected in this compilation.)

Editor: Roberto Buccione

1st Editorial Decision 16 September 2015

Thank you for the submission of your manuscript to EMBO Molecular Medicine. We have now heard back from the three Reviewers whom we asked to evaluate your manuscript.

As you will see, the Reviewers find merits in your manuscript but raise significant issues. Based on our discussion here, the main concern is that, in aggregate, the Reviewers find that the conclusions deriving from the drug efficacy study are not sufficiently supported by the experimentation. The reasons for this are clearly detailed in the evaluations. Another shared concern is on the quality and depth of exome analysis.

The Reviewers also list several other items of concern, which do not require further discussion here, as they are clearly explained and straightforward.

I would like, however, to highlight 2 specific items mentioned by Reviewer 3. S/he challenges the conclusions drawn on NF1-associated vs. sporadic MPNST based on the models and data presented. Reviewer 3 also notes that in addition to the insufficient experimental support for the drug study, as mentioned above, it would also appear that the models do not predict the results obtained in the clinic (albeit with very small numbers). Clearly, these are both crucial points.

In conclusion, while publication of the paper cannot be considered at this stage, given the potential interest of your findings and the fact that the Reviewers, although critical, were globally positive, we have decided to give you the opportunity to address the above concerns.

We are thus prepared to consider a substantially revised submission, with the understanding that the Reviewers' concerns must be addressed with additional experimental data where appropriate and that acceptance of the manuscript will entail a second round of review. This also includes significantly upgrading the strength and conclusiveness of the drug studies. Please note that we consider this aspect of your work to be of prime importance and without it we will not be able to move forward with your manuscript.

I understand that to address the above might entail a significant amount of time, additional work and experimentation and might be technically challenging, I would therefore understand if you chose to rather seek publication elsewhere at this stage. Should you do so, we would welcome a message to this effect.

Please note that it is EMBO Molecular Medicine policy to allow a single round of revision only and that, therefore, acceptance or rejection of the manuscript will depend on the completeness of your responses included in the next, final version of the manuscript.

As you know, EMBO Molecular Medicine has a "scooping protection" policy, whereby similar findings that are published by others during review or revision are not a criterion for rejection. However, I do ask you to get in touch with us after three months if you have not completed your revision, to update us on the status. Please also contact us as soon as possible if similar work is published elsewhere.

I look forward to seeing a revised form of your manuscript in due time.

***** Reviewer's comments *****

Referee #1 (Comments on Novelty/Model System):

This is a well written study describing a preliminary study on a limited number of orthoxenografts. The molecular genetic characterization of graft tumors is performed in a very comprehensive way. The orthoxenograft model is quite interesting but didn't allow concluding on the drug efficacy. This study is more a description and genetic validation of an orthoxenograft model of MPNST than a pharmacological study.

Referee #1 (Remarks):

This study by Castellsague et al. describes the establishment of 5 patient-derived MPNST orthoxenograft models and preliminary results of pre-clinical pharmacological testing on these models.

This is a well written study describing a preliminary study on a limited number of orthoxenografts. The molecular genetic characterization of graft tumors is performed in a very comprehensive way. The orthoxenograft model is quite interesting but didn't allow concluding on the drug efficacy. This study is more a description and genetic validation of an orthoxenograft model of MPNST than a pharmacological study.

Remarks:

Citations and table legends format should be homogenised.

Introduction:
Existence of clinical trails using several pharmacological agents targeting the RAS-MAPK and the PI3K-mTOR pathways should be specified.

More information should be given concerning the S462 cell line.

Lifetime risk is 8-13% in Evans et al. article.

Mat&Met: some points need to be clarified and specified

Primary tumors and cell lines
´fresh primary MPNST ª : From how many patients?
´culture medium ª: Which one?

Human MPNST implantation
´Once orthotopic tumors had reached a volume of 1000-1500 mm3ª. This volume seems quite high, as compared to other studies...
´ 6 orthoxenografts ª 4 MPNST + 1 cell line = 5?
How many mice were generated for each tumor? What was the size of tumors at each passage (explants and transplant)?

Immunohistochemistry
Antibodies dilutions should be specified.

Exome sequencing
"small insertions and deletions were not counted" Why??

Drug treatment
Treated mice groups should be specified: which stage, which passage?
´ Seven treatment regimens were tested ª Please, clearly specify the seven treatments.
Each drug is dissolved in a different solvent. Was a placebo mouse injected with each solvent?
"Overall treatment time varied slightly between experiments (12-25 days), according to the intrinsic differences in tumor growth, and was always marked by the tumor size in the matching control group." Sacrifice times should be specified. How were tumors size measured?

Mat&Met should include a paragraph dealing with micrometastases research. Was this research performed in all mice? How? Only in lung and liver?

Results

Figure1 legend is not clear. What do PT and OT mean? It is claimed that "1 mm3 of the primary tumor... were graft" versus 2-3 mm3 in the Mat&Met.

Figure 2B legend: ´ p53 is shown for primary tumors ª but Ki67 is shown on pictures.

Human stroma is lost after engrafment
Previous studies (notably from Luis Parada group) dealing with the importance of tumor environment in NF1 tumorigenesis should be cited in the introduction section.

Figure 3A: Please explain the bar colour code in the legend (green?)
Figure 3B: Sanger sequencing is not described in the Mat&Met section. Why was the c.350T>A mutation not found with 50% WT and 50% mutated allele frequencies in blood? Which OT was sequenced?

Molecular validation
It is claimed that ´ global view of the genomic profiles showed high degree of similarity between primary tumour et 4 orthoxeno ª but also that ´ fewer alteration in primary tumour".

It seems difficult to compare CNVs profile of MPNST A that only shows 20% of tumor cells, with the other tumor profiles.

No profile for the graft cell line?

Exome sequencing
There was ~twice more covered regions in MPNST-NF1 vs. MPNST-SP: isn't it difficult then to compare the number of mutations?

Expression analysis
No expression profile for NF1 MPNST 001 PT and OT P4?

Pre-clinical
How many mice per group?
´ intraperitoneal adm of apa caused signif tumor reduction in all models ª : no results shown for MPNST-NF1-S462.
Only 1 NF1 patient was tested.

Discussion
The very limited number of xenograft and treated mice only allows very speculative conclusions when (i) comparing genetic alterations in sporadic versus NF1-associated MPNSTs (only 1 NF1 MPNST was tested) and (ii) describing putative drug efficacy.

The title: "Effective sorafenib treatment response in a panel of genomically-characterized malignant peripheral nerve sheath tumor orthoxenograft models" should be modified as no affirmative conclusion can be drown from this very limited panel.

Referee #2 (Remarks):

Castellsague et al

The manuscript by Castellsague et al describes experiments designed to determine if it is possible to create human malignant peripheral nerve sheath tumor (MPNST) orthotopic xenografts in nude mice. The authors succeed in a few NF1-associated and sporadic MPNSTs. The rate of success was high. The authors characterize the genomic signature of the MPNSTs before and after serial passage in mice using SNP arrays, gene expression analyses and whole exome sequencing. They present evidence for loss of human stromal cells with passage. The paper generally iswell written and provides high quality data. The work is useful given that almost nothing has been described on primary MPNST xenografts in immunodeficient mice. The paper could provide a useful resource to the filed if the xenografts are provided to the research community. Specific comments follow:

1. The orthotopic injection procedure is not described well enough
2. The authors make too much of a limited comparison between NF1 and non-NF1 related MPNST genetic characteristics
3. The loss of MPNST tumor stroma over passage generations, is very interesting. Can the authors do marker studies by IHC to determine which stromal cells are present in early passage orthiografts that are lost later?
4. The dose and schedules for drug treatment are not really justified. How were they chosen? Can the authors provide any data indicating that the drugs are hitting the intended molecular targets? That is, pharmacodynamics studies?
5. The authors should also reference Watson et al. Oncotargt, 2014.
6. Can the authors comment on the low depth of coverage for the whole exome studies? Is it possible mutations and subclones are being missed? Also, access to the raw data for exome re-sequencing is vital for this paper. A supplementary table on all variants detected is vital. The authors describe the variants detected by whole exome sequencing as "somatic", but this is not justified as the authors did not sequence germline DNA from each patient. The "variants" should be compared to dbSNP as part of this analysis. Clear loss of function mutations should be reported too.
7. Supplementary Fig. 4 is hard to interpret visually.

Referee #3 (Remarks):

In the manuscript, "Effective treatment response in a panel of genomically-characterized malignant peripheral nerve sheath tumor orthoxenograft models," Castellsague et al characterize new MPNST orthotopic models from 1 NF1 patient and 2 sporadic MPNST patients. MPNST is a deadly, rare disease that has been difficult to develop therapies for, so this manuscript reports a valuable new tool for developing MPNST therapy. The authors carefully characterize the model histologically and molecularly, although they overinterpret their results on differences between NF1-associated and sporadic MPNST due to the low number of independent samples examined.

Major Issues:
The abstract is misleading, because the five models that have been generated are not truly independent (all of the NF1 models are from the same patient). Furthermore, one of the five models is derived from a cultured cell line (S462) and is not considered a patient-derived xenograft. The description of the five models should be rephrased to make clear that they are not all biologically independent.

For the histological validation, the figures are too small, and too low resolution, to verify the MPNST morphology of the cells in the tumor. At the very least, the 400X panel should be made the full size. Lower magnification photos could be moved to supplemental figures. Are mitotic figures present in these tumors? The inset would be better used showing clear morphology of a couple of cells or mitotic figures.

The authors should report the ethnicity of the patients from which the samples are collected in Table 1, as this could become an important confounding factor in the study of MPNSTs in the future.

In most of the histological sections shown, the grafted tumor appears to be more densely cellular than the patient sample, suggesting perhaps a loss of extracellular matrix (ECM) material. Immunohistochemical staining for collagen IV and laminin should be examined to determine if the tumor cells produce similar levels of ECM in the grafts as the in situ tumors.

The metastatic phenotype is particularly interesting and valuable to researchers developing therapies. The authors should add photos of the late stage micrometastases to Figure S2, and make clear which models showed the metastases. Was this phenotype specific to the sporadic MPNST models? The supplemental figure is pixelated and the histology of the metastasis cannot by reviewed. A better resolution figure is needed. The metastases should also be stained for S100 to verify their MPNST origin. With the current data as presented it is not possible to exclude that these are infections in the lung.

For the molecular analysis of SNPs, the authors analyzed samples at different times on three different Illumina chips. This has the potential for introducing batch effects that may be interpreted as differences between NF1 and sporadic tumors, or in situ tumors and xenografts. Which samples were run in which batches? Was there any common sample run on all chips to normalize batch effects? How were losses of SNPs handled between different platforms? The authors should also include the sex chromosome data and mitochondrial data where available.

For the gene expression analysis, it is not clear why the NF1-001 patient sample was not included. This would provide important information for using this model. Is sample available to include in this analysis?

While the drug testing presented is promising, it should be noted that sorafenib and the rapamycin analog have already been tested in soft tissue sarcomas, including MPNST, without clear efficacy. In the clinical trial of sorafenib (NCT00245102), 12 MPNST patients were treated and none had a complete or partial response. In a clinical trial of temisirolimus (Okuno et al 2011 Cancer 117:3468) only 5% of sarcoma patients showed a response, and the MPNST patient in the study did not respond. While it is still possible that combinations of these therapies would be beneficial, the results shown in Figure 7 suggest that these models may not predict results in the clinic. The authors

should thoroughly discuss what is known about the tested drugs in sarcoma clinical trials in the discussion section.

Throughout the manuscript the authors draw preliminary conclusions on the comparison of NF1-associated MPNSTs and sporadic MPNSTs. Given that the 2 NF1 patient derived xenografts come from the same patient, it is hard to say whether the NF1 tumors are clustering because they are genetically related, because they come from a young patient, or because they are NF1 associated. These preliminary conclusions on differences between sporadic MPNST and NF1 MPNST should be removed from the abstract, from the molecular genomic results (SNP, exome seq, and gene expression), the entire section comparing sporadic vs NF1-associated models, and the explanation of the paper. While it is useful to point out differences between the 5 models, there are not enough samples in any one group to conclude these differences correlate to NF1 disease, the mutation in the NF1 gene, the age of the patient, or loss of linked genes on chr 17 during LOH. All of these possibilities should be included in the discussion section.

Additional details should be added to the Material and Methods section:
What are the conservation protocols for the patient tumors? Please describe in more detail or cite another reference.
What committee approved the human tissue study?
For the grafting into the sciatic nerve, please provide more details. Was the piece of tumor placed along side the nerve? Was the epineurium breached? Why were the S462 cells injected into the muscle and not into the sciatic nerve?
How were drug formulations chosen? Had these been used previously in preclinical mouse studies (please cite references).

Minor Issues:
Turk et al J Vis Exp. 2011 Mar 7;(49). pii: 2558. doi: 10.3791/2558 should be added to the references on pg 5 as a orthotopic xenograft generated from an MPNST cell line.

Figure 3 panels are out of order in the manuscript. Figure 3A could be moved to the bottom for Figure C and other panels moved accordingly so the figures are in the order cited in the manuscript. In the current Figure 3A the green bar and the red line are not defined in the figure legend.

Pg 8, 2nd new paragraph: Change "composed by" to "composed of."

Pg 10, 1st new paragraph: Change "Table SI" to "Table S1." Should supplemental figure 5 be cited here?

Make paragraph indenting consistent throughout.

The S462 cell line was generate by Lan Kluwe's group and is described in Frahm et al Neurobiology of Disease (2004) 16:85-91 (patient 6). The citation should be added to the manuscript.

Pg 21, 3rd paragraph: Change "tight" to "thigh" in the 3rd line.

Supplementary Figure S2 legend: The second line appears to be a fragmented sentence. Do the authors mean the tumors of the orthotopically implanted mice or the lungs? It doesn't make sense to say the "mice were removed by surgery." Is the histology of the middle panel of the tumor or of the lung or liver?

Summary: The authors present well characterized models for drug testing of human MPNST that are desperately needed in the field. However, this manuscript has substantial flaws in the interpretation of the data. If these flaws can be remedied, the manuscript may be suitable for publication, although re-review would be important.

1st Revision - authors' response 04 January 2015

We have addressed each of the questions raised by the referees and hope that the revised version meets their requirements and is suitable for publication in your journal. We would like to thank the referees and the editorial board for providing us with useful comments and suggestions that have improved the quality of our manuscript. Below are point-by-point answers to each of the questions and comments raised in the revision process. Note that the comments of the editorial board or the referees are given in italic type.

Editorial comments

As you will see, the Reviewers find merits in your manuscript but raise significant issues. Based on our discussion here, the main concern is that, in aggregate, the Reviewers find that the conclusions deriving from the drug efficacy study are not sufficiently supported by the experimentation. The reasons for this are clearly detailed in the evaluations. Another shared concern is on the quality and depth of exome analysis. I would like, however, to highlight 2 specific items mentioned by Reviewer 3. S/he challenges the conclusions drawn on NF1-associated vs. sporadic MPNST based on the models and data presented. Reviewer 3 also notes that in addition to the insufficient experimental support for the drug study, as mentioned above, it would also appear that the models do not predict the results obtained in the clinic (albeit with very small numbers). Clearly, these are both crucial points.

We have addressed the three main issues raised by the reviewers and the editorial board as follows:

1) In relation to the drug treatment performed using the models presented:

- New data have been included that support the tumor response to drug treatments. A table has been added, summarizing the histopathological response in post-chemotherapy tumor masses, together with a supplementary figure showing a better histological characterization of orthoxenografts (% of necrosis and number of mitosis) after each treatment (Table 3 and Supplementary Figure S6). The main message is that, looking at response to treatment in the models generated, the highest efficacies are reached when using combined treatments, especially those including sorafenib.

- We have also included new data to better characterize long-term responses to drug treatment. A panel summarizing the time to tumor relapse after chemotherapy has been added to Figure 7 (Figure 7B).

- Distal dissemination properties have been better investigated. Lung, brain and liver from sacrificed mice were histologically analyzed for the presence of micrometastases and a Supplementary Figure has been included. Immunohistochemistry in MPNST metastasis for Vimentin, CD34, S100 and Ki-67 has been included. Data on dissemination capabilities over a longer time frame (4-6 months after tumor removal) have also been included.

- A paragraph discussing our preclinical results in the context of the clinical trials performed so far has been added to the Discussion section. There is still very little data from clinical trials on MPNST, and only single drug therapies have been trialed thus far. Our preclinical results, although still limited, are not in disagreement with the clinical data, since they point to combined therapies as the best chemotherapy schemes.

- Finally, we have changed the title of the manuscript as suggested by one of the referees. The title of the manuscript now reads: "*Comprehensive establishment and characterization of orthoxenograft mouse models of malignant peripheral nerve sheath tumors for personalized medicine*". We believe that this title better describes the whole work and reflects the referees' comments on the importance of the description and genetic validation of the newly generated orthoxenograft MPNST mouse models, rather than the pharmacological study. The referees believe that our models will provide a useful resource to the field, and the new title more clearly reflects this.

2) In relation quality and depth of exon analysis:

Quality of exome analysis has been better described and a supplementary table with a list of all variants, including small indels and point mutations, had been added. Exome quality meets current standards. We have clarified the criteria and the use of well-covered regions common to all samples for comparing orthoxenografts to primary tumors for validating the models. We also have stressed that exome data have mainly been used for validation purposes and not for biological characterization.

<u>3) In relation to the sporadic *vs.* NF1 model comparison:</u>

As suggested by the three referees, we have toned down discussion of the differences observed between sporadic and NF1-associated MPNST: the specific section covering this topic has been removed from the Results section, and it is no longer mentioned in the Abstract. The overall importance of this comparison has been toned down throughout the manuscript; the matter is now presented in a more descriptive manner, and no hard conclusions are stated.

Answer to Referee #1

Citations and table legends format should be homogenised.

All the citations and table legends have been homogenised.

Introduction: Existence of clinical trails using several pharmacological agents targeting the RAS-MAPK and the PI3K-mTOR pathways should be specified.

The following paragraph has been added to the Introduction section: "Two recent phase II clinical trials assessed the monotherapy activity of sorafenib or rapamycin analogs (temsirolimus) in patients with different types of sarcoma, including MPNST (Maki et al., 2009; Okuno, 2011). In general, no objective responses were observed in this subset of patients when using a single drug treatment."

More information should be given concerning the S462 cell line.

A detailed description of cell line S462 has been added to the Material and Methods section: "S462 belongs to an NF1 patient carrying the c.6792C>A nonsense mutation in exon 37. This patient developed a grade IV MPNST on the thigh at age 19. The MPNST carried LOH in the *NF1*, *TP53* and *CDK2NA* genes. Cell line establishment was described in Frahm et al., *Neurobiology of Disease 16* (2004)."

Lifetime risk is 8-13% in Evans et al. article.

Lifetime risk has been corrected accordingly.

Mat&Met: some points need to be clarified and specified. Primary tumors and cell lines «fresh primary MPNST»: From how many patients? «culture medium »: Which one?

Four fresh tumors were obtained from three patients: two independent MPNSTs from one NF1 patient and two independent MPNSTs each from a different patient. After surgery a piece of each tumor was immersed in DMEM, 10% FBS culture medium.

The description of tumors and patients in the Material and Methods section has been clarified and now reads: "Four fresh primary MPNST from three different patients were identified and removed at the Sarcoma Clinical Unit (UFTOS) of Bellvitge Hospital (HUB) and the Catalan Institute of Oncology (ICO), both institutions located on the IDIBELL campus. Two independent MPNST were from one NF1 patient and the other two were from two different sporadic patients. After surgery, the tumor was sent to our pathology service where it was analyzed following standard protocols. Simultaneously, a piece of each tumor was stored in DMEM, 10% FBS culture medium at room temperature before being sent to our molecular unit. Once in our laboratory the tumor was divided into sections, processed and preserved in order to have material for different purposes. Small pieces of each tumor were directly frozen in liquid nitrogen so that DNA, RNA and/or protein could be obtained when needed. Small pieces were frozen in appropriate culture media so that cell culture experiments or mice engraftments could be performed. Informed consent was obtained from all subjects and the study received the approval of the IDIBELL Ethics Committee."

Human MPNST implantation «Once orthotopic tumors had reached a volume of 1000-1500 mm3». This volume seems quite high, as compared to other studies...

The referee is right to say that a tumor mass of 1000-1500 mm^3 is a large mass, but this size is particularly large when dealing with subcutaneous engrafted tumors, since these tumors are easily and directly measured. However, in orthotopic-grafted tumors, and especially in our MPNST models, we have to take into account that the tumors are inside the muscle and are more difficult to measure, since they are detected once they have already grown to a considerable size. In addition, the measurement is always overestimated, since muscle and skin are also taken into account when measured with the caliper instrument used for this purpose. For this reason, subcutaneous tumors tend to be necrotic when they reach this size, but this volume is perfectly workable in our

orthotopic-grafted models. This can be clearly observed in the new Table 3, where a low level of necrosis is observed in non-treated tumors at the moment of mouse sacrifice, when tumors are around 2000-2500 mm^3 in volume. We have directly observed that tumors of this size do not dramatically affect mouse welfare. Lastly, we would like also to emphasize that our procedure for MPNST orthoxenografts was approved by our campus Animal Ethics Committee and complied with AAALAC procedures (this has been also added to the Material and Methods section).

« 6 orthoxenografts » 4 MPNST + 1 cell line = 5?

This has been clarified in the Material and Methods section. For the first NF1 model established (MPNST-NF1-001) we analyzed two independent engraftments at passages 1 and 4, which is why the total number of orthoxenografts is six instead of five in some of the molecular analyses. This has been explained more clearly in the Material and Methods section.

How many mice were generated for each tumor? What was the size of tumors at each passage (explants and transplant)?

Model establishment and perpetuation:
- To generate the model, a small piece of the primary tumor (2-3 mm^3) was grafted into a minimum of three mice.
- From passage #2 on, at least three mice were implanted each time to expand the orthoxenografted tumor.
- In general, when tumors reach a size of 1000-1500 mm^3, mice were sacrificed; tumors were harvested, cut into small fragments (2-3 mm^3) and implanted in three new mice.
- In order to perpetuate the model, between seven and ten passages were performed.

Immunohistochemistry. Antibodies dilutions should be specified.

We have specified all antibody dilutions except for the ready to use (RTU) products, since in these cases this is proprietary information of the respective companies. In these cases, following other examples in the literature, we used the IR/IS of the RTU product.

Therefore, the paragraph now reads:

"The primary antibodies vimentin (1:500, IR630, DAKO), Desmin (IR606, DAKO), Actin (1:50, M0851, DAKO), EMA (1:200, IR629, DAKO), CD34 (IR632, DAKO), S100 (IS504, DAKO), P53 (IR616, DAKO) and Ki-67 (1:75; M7240, DAKO) were incubated overnight at 4ºC following the manufacturer's guidelines."

Exome sequencing: "small insertions and deletions were not counted" Why??

We have added a supplementary table (Supplementary Table 1) with a list of all the variants, including point mutations and small indels, present in well-covered regions common to all samples derived from the same patient (WCR-PRS). However, since current indel calling algorithms are known to have a high false positive error rate, for the purposes of comparison and validation only point mutations were taken into account. It is worth noting that indels represent only a small proportion of the total number of variants identified.

Drug treatment. Treated mice groups should be specified: which stage, which passage?

In order to prepare each drug treatment, an early-passage (P2-P4) orthoxenograft tumor had to be expanded. To do this, each tumor was implanted in five mice. When tumors reached a minimum size of 1000-1500 mm^3, mice were sacrificed, tumors were harvested and cut into small fragments, and the tumor fragments were grafted into 60-80 mice, depending on the size of the experiment.

« Seven treatment regimens were tested » Please, clearly specify the seven treatments.

The seven treatments have been clearly specified in the drug treatment part of the Material and Methods section, as follows:

"Seven treatment regimens were tested: 1) doxorubicin; 2) intraperitoneal rapamycin; 3) oral rapamycin; 4), sorafenib; 5) doxorubicin plus oral rapamycin; 6) doxorubicin plus sorafenib, and 7) oral rapamycin plus sorafenib."

Each drug is dissolved in a different solvent. Was a placebo mouse injected with each solvent?

We have clarified this point in the drug treatment part of the Material and Methods section by adding the following sentence: "A mouse control group receiving no drug was used for each drug

treatment experiment. In the first drug experiment, using mice MPNST-NF1-001, two additional control groups were treated with the two vehicles for oral rapamycin and sorafenib administration. No significant differences in tumor response were observed between the different vehicles relative to the untreated group. Thus, to simplify the presentation of data, we have included only one control group per model, which corresponds to the untreated animals."

"Overall treatment time varied slightly between experiments (12-25 days), according to the intrinsic differences in tumor growth, and was always marked by the tumor size in the matching control group." Sacrifice times should be specified. How were tumors size measured?

Sacrifice times have been added to the Material and Methods section. Tumors were measured using a caliper (this is now stated). The text now reads: "Briefly: MPNST-NF1-001 treatments lasted 12 days, MPNST-NF1-002 treatments lasted 22 days, MPNST-SP-001 treatments lasted 14 days, MPNST-SP-002 treatments lasted 25 days, and MPNST-NF1-S462 treatments lasted 19 days. After treatment initiation, tumors were measured using a caliper every 2-3 days […]"

Mat&Met should include a paragraph dealing with micrometastases research. Was this research performed in all mice? How? Only in lung and liver?

We have investigated in more depth the presence of metastases in all of the samples collected. We now present additional data and new figures illustrating our results. As suggested by the reviewer, a paragraph about the metastases has been added to the Material and Methods section, which now reads as follows: "To investigate the capability of the orthoxenograft tumors to disseminate in mice, the lungs, livers and brains of 45 animals were histologically examined by H&E staining for the presence of micrometastases". Additionally, some of the metastases have been further characterized by immunohistochemistry.

Results. Figure1 legend is not clear. What do PT and OT mean? It is claimed that "1 mm3 of the primary tumor... were graft" versus 2-3 mm3 in the Mat&Met.

The figure legend has been clarified and corrected as suggested.

Figure 2B legend: « p53 is shown for primary tumors » but Ki67 is shown on pictures.

The referee is right, this has been changed accordingly.

Human stroma is lost after engrafment. Previous studies (notably from Luis Parada group) dealing with the importance of tumor environment in NF1 tumorigenesis should be cited in the introduction section.

The importance of the tumor environment has been explained and cited in the Introduction.

Figure 3A: Please explain the bar colour code in the legend (green?)

This has been explained.

Figure 3B: Sanger sequencing is not described in the Mat&Met section.

Sanger sequencing is now described in this section.

Why was the c.350T>A mutation not found with 50% WT and 50% mutated allele frequencies in blood? Which OT was sequenced?

The electropherogram of a Sanger sequence presenting a single nucleotide change in heterozygosity is not always seen as a balanced peak pair and can also appear somewhat imbalanced, as in this case. It has been described that the specific outcome for a given peak pair is typically highly reproducible and depends on the local sequence context (Applied Biosystems, Technical Tips Protocol). Moreover, this mutation was also detected when performing exome sequencing by NGS in the patient's blood sample and was observed in approximately half of the reads, as expected.

We sequenced an OT passage-1 tumor (which is now described in the figure legend).

Molecular validation. It is claimed that « global view of the genomic profiles showed high degree of similarity between primary tumour et 4 orthoxeno » but also that « fewer alteration in primary tumour". It seems difficult to compare CNVs profile of MPNST A that only shows 20% of tumor cells, with the other tumor profiles.

This primary tumor (MPNST-NF1-01) contained a very high percentage of 2n cells, which made it difficult for the copy number calling algorithm to report all of the alterations present in the aberrant

cells; fewer alterations were ultimately reported. However, a visual inspection of the raw data (see Figure 4) shows that almost all alterations identified in orthoxenografts were in fact also present in the aberrant fraction of the primary tumor. In the other primary tumors (see Figure S4) this problem is minimized and the comparison is easier due to the lower number of 2n cells present in the rest of the primary tumors.
Now the paragraph reads as follows:
"*A global view of the genomic alteration profiles showed a high degree of similarity between the primary tumor and the 4 derived orthoxenografts. In this case, due to the high proportion of non-altered stroma cells in the primary tumor sample, the raw data were strongly biased towards a diploid heterozygous genome, hence the variant calling algorithm used reported fewer alterations in the primary tumor than in orthoxenografts. However, visual inspection of the raw data revealed that almost all alterations identified in orthoxenografts were present in primary tumors (see Figure 4). Furthermore, these differences were not present in the rest of the primary tumor vs. orthoxenograft comparisons, since these tumors contained a lower proportion of 2n cells (Figure S4A and S4B).*"

No profile for the graft cell line?

The SNP-array profile of the grafted cell line is explained in the section "Cell line *versus* primary tumor orthoxenograft models" and illustrated in Figure 8.

Exome sequencing. There was ~twice more covered regions in MPNST-NF1 vs. MPNST-SP: isn't it difficult then to compare the number of mutations?

All comparisons between tumors were made taking into account ONLY the regions with sufficient coverage (≥20x) in ALL of the tumors. These well-covered regions were termed WCR. This correction for differences in coverage makes the comparison feasible and reliable, since the exact same genomic regions were compared between tumors.

Expression analysis. No expression profile for NF1 MPNST 001 PT and OT P4?

There were no data for these two samples: a) there was no mRNA for NF1 MPNST 001 PT; b) the expression-array failed for SP-001 OT P4 due to poor mRNA quality.

Pre-clinical. How many mice per group?

The number of mice for treatment is described in the Material and Methods section: 7-10 per treatment. We have added a sentence to the Material and Methods section to clarify the number of mice used for the different drug assay experiments. The apparent confusion came from the previous version of the paper, in which we did not include the results for long-term response evaluation in mice. Long-term response studies should add between 3-5 mice per treatment; these mice are kept at the end of chemotherapy until a tumor size of 1500-2000 mm^3 is reached. In summary, for a *classical* experiment including short- and long-term responses for seven different treatment schemes, a total of 50-70 mice were implanted. Depending on the success and homogeneity of tumor growth at the moment of mouse randomization, to allocate animals to the different treatment groups, the final number was adjusted so that both short- and long-term responses could be assessed.

« intraperitoneal adm of apa caused signif tumor reduction in all models » : no results shown for MPNST-NF1-S462.

The referee is right; no results are shown for this treatment/model, due to a technical problem with the number of mice that prevented us from performing this treatment in model of cell line S462. This is now stated in the text.

Only 1 NF1 patient was tested.

Yes, the referee is right. We have clarified this in the text. We used two independent NF1 MPNST tumors (clearly independent, according to the genomic characterization) but from the same NF1 patient.

Discussion. The very limited number of xenograft and treated mice only allows very speculative conclusions when (i) comparing genetic alterations in sporadic versus NF1-associated MPNSTs (only 1 NF1 MPNST was tested) and (ii) describing putative drug efficacy. The title: "Effective sorafenib treatment response in a panel of genomically-characterized malignant peripheral nerve sheath tumor orthoxenograft models" should be modified as no affirmative conclusion can be drown from this very limited panel.

(i) We agree with the referee that general conclusions cannot be drawn from the limited number of NF1 vs. sporadic models generated. However, we would like to clarify that although both NF1-related MPNST models were from the same NF1 patient, the tumors were clearly independent (as demonstrated by the genomic characterization). However, as suggested by various referees, we have toned down discussion of the differences observed between sporadic and NF1-associated MPNST: the specific section covering this topic has been removed from the Results section, and it is no longer mentioned in the Abstract. The overall importance of this comparison has been toned down throughout the manuscript; the matter is now presented in a more descriptive manner, and no hard conclusions are stated.

(ii) Regarding the drug treatment results obtained, the conclusions are always constrained by the panel of models analyzed. Thus, since we have observed an effective treatment using sorafenib, especially in a combined regimen, conclusions—thought limited—can be drawn and presented. However, in light of the referees' comments, and taking into account the body of work presented, we have changed the title of the manuscript to read as follows: *Comprehensive establishment and characterization of orthoxenograft mouse models of malignant peripheral nerve sheath tumors for personalized medicine*.

Answer to Referee #2 (Remarks):

1. The orthotopic injection procedure is not described well enough

As suggested by the reviewer, we provide a better description of the methodology for orthotopically injecting the S462 cell line close to the sciatic nerve in mice.

In the material and methods section a sentence has been added to the manuscript, as follows: "To establish the orthoxenograft model from the S462 NF1-MPNST cell line, we injected 0.3 ml of the cell suspension ($3x10^6$ cells) with a needle directly in the upper thigh muscle using Matrigel (a solubilized tissue basement membrane matrix rich in extracellular matrix proteins). This enabled tumor growth around the epineurium."

2. The authors make too much of a limited comparison between NF1 and non-NF1 related MPNST genetic characteristics

We agree with the referee that the limited number of models does not allow general conclusions to be reached on the comparison between NF1 and sporadic models. As such, we have avoided drawing general conclusions and limit our results to describing the differences between the models. As suggested by the referee, we have therefore removed the section "Sporadic vs. NF1-associated orthoxenograft mouse MPNST models" and adapted the content of the Abstract and the main text dealing with this comparison.

3. The loss of MPNST tumor stroma over passage generations, is very interesting. Can the authors do marker studies by IHC to determine which stromal cells are present in early passage orthografts that are lost later?

To investigate the presence of mouse stroma in the grafts we performed staining for two major cell types: endothelial cells (CD34 antibody) and fibroblast (smooth muscle actin-SMA antibody). We tried to use species-specific antibodies, but due to the high degree of sequence identity between mouse and human proteins, this was not always possible. The staining with anti-human CD34 clearly detected human-derived endothelium in primary tumors but not in orthoxenograft tumors, as shown in Figure 3. However, when using anti-human SMA, although primary tumors exhibited a higher staining intensity, some background staining was also detected in the xenograft, which impeded a clean experimental response. Consequently, the results for SMA staining were omitted, but are now attached below.

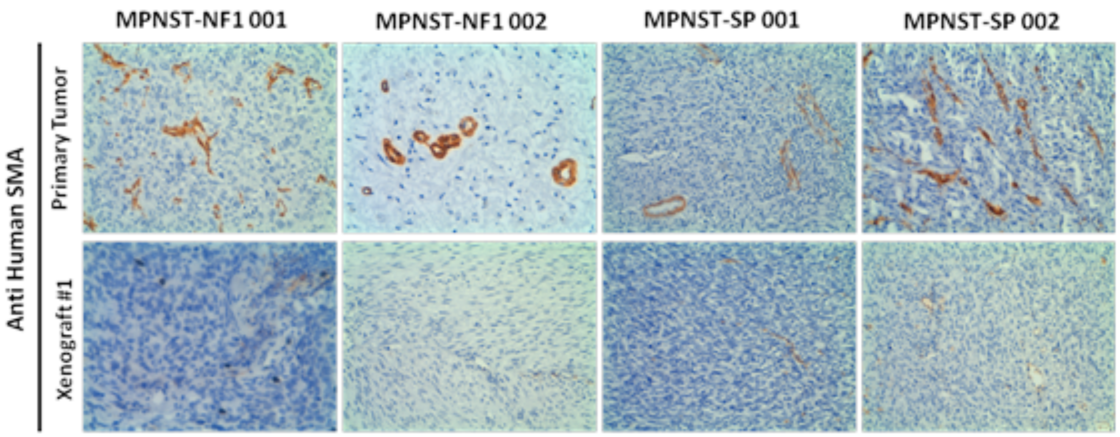

4. The dose and schedules for drug treatment are not really justified. How were they chosen?

The dose and schedules for each drug have been explained in more detail in the Material and Methods section. References from previous studies were used to establish our drug treatment protocol and have been added to the manuscript, as follows: "Doxorubicin dose was chosen on the basis of studies in which intraperitoneal administration at 8 mg/kg was tested in a xenograft model derived from a MPNST cell line (Johansson et al., 2008). Rapamycin administered intraperitoneally at 5 mg/kg was used previously in a genetically engineered MPNST mouse model (Johannessen et al., 2008), and the sorafenib dose was chosen on the basis of preclinical studies in which daily oral administration of Sorafenib at 30-60 mg/kg was tested in several tumor models (Wilhelm, 2004)."

Can the authors provide any data indicating that the drugs are hitting the intended molecular targets? That is, pharmacodynamics studies?

We did not perform any systematic pharmacodynamics study because it was out of the scope of our work. The drugs that we used are well known and have been well characterized in terms of pharmacodymanics and mode of action. However, data collected in a non-systematic way during the drug treatments point to a correct and specific action of the compounds used. For instance, we performed a Western blot analysis for the MPNST-NF1-001 model, using an antibody recognizing the activated (phosphorylated) form of the mTOR downstream effector protein, ribosomal S6. We found that after rapamycin treatment, MPNST-NF1-001 orthoxenograft tumors had lower levels of phospho-S6 than untreated mice or mice treated with doxorubicin (see accompanying figures).

The figure below corresponds to a Western blot analysis of lysates from MPNST-NF1-001 tumors using the following antibodies: anti-phospho S6K1, anti-total S6 and Tubulin. CNT: control group, DOX: doxorubicin-treated mice and RAP: rapamycin-treated mice.

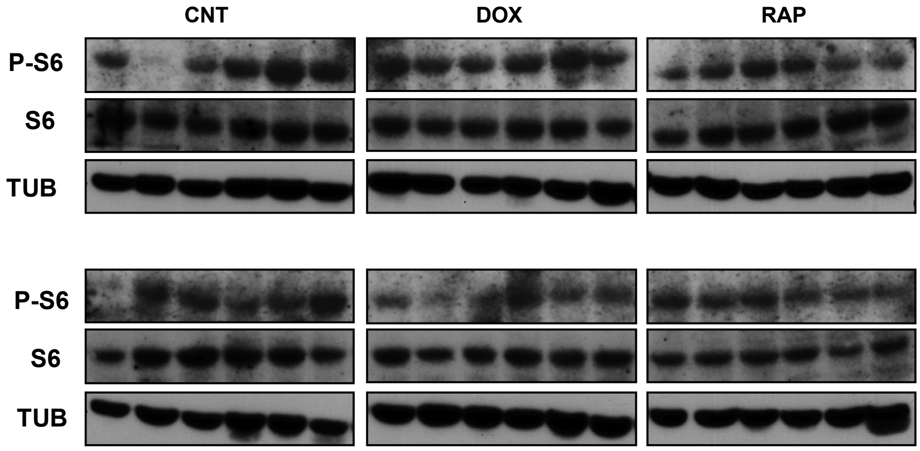

© EMBO

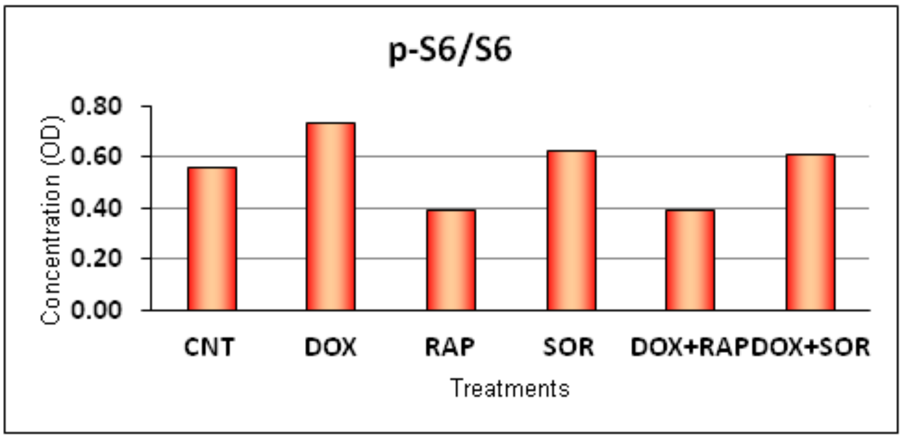

5. The authors should also reference Watson et al. Oncotarget, 2014.

This reference has been added.

6. Can the authors comment on the low depth of coverage for the whole exome studies? Is it possible mutations and subclones are being missed? Also, access to the raw data for exome re-sequencing is vital for this paper. A supplementary table on all variants detected is vital. The authors describe the variants detected by whole exome sequencing as "somatic", but this is not justified as the authors did not sequence germline DNA from each patient. The "variants" should be compared to dbSNP as part of this analysis. Clear loss of function mutations should be reported too.

The mean exome coverage for all the samples analyzed is 64X. We believe that this is a reasonable coverage for the validation analysis performed here, although it is true that mutations and subclones may be missed, as is true of almost any other exome performed with current depth of coverage standards. We would like to clarify that in this work we have used exome sequencing data (as well as all the other genomic techniques used) only to validate the developed models, by comparison with the respective primary tumors. Thus, we have not used the specific mutations identified to draw any conclusions on the biology of the tumors and models developed, since this falls outside the scope of the paper. Nonetheless, we have added an additional supplementary table (Supplementary Table 1) with a list of all variants, including point mutations and small indels, present in well-covered regions common to all samples derived from the same patient (WCR-PRS). Since raw exome data can be used to identify the tissue donors, they cannot be publicly released. We are studying the feasibility of depositing these data to the EGA, where they would be available upon request.

We have clarified in the manuscript that, for all patients, constitutional DNA was also exome-sequenced. The comparison of exome data from constitutional DNA with matched tumor DNA allowed us to identify somatic variants. As stated in the Materials and Methods: "Common variants, defined as those present in dbSNP135 with a minor allele frequency >1%, were filtered out." Putative effects at the protein level are also included in Supplementary Table 1.

7. Supplementary Fig. 4 is hard to interpret visually.

To facilitate the visual interpretation of the Circos plots, we have divided the Figure in two pages, the first showing the two NF1 models and the second showing the sporadic models.

Answer to Referee #3

The abstract is misleading, because the five models that have been generated are not truly independent (all of the NF1 models are from the same patient). Furthermore, one of the five models is derived from a cultured cell line (S462) and is not considered a patient-derived xenograft. The description of the five models should be rephrased to make clear that they are not all biologically independent.

We agree with the referee. All of these aspects have been clarified in the Abstract.

For the histological validation, the figures are too small, and too low resolution, to verify the MPNST morphology of the cells in the tumor. At the very least, the 400X panel should be made the full size. Lower magnification photos could be moved to supplemental figures. Are mitotic figures present in these tumors? The inset would be better used showing clear morphology of a couple of cells or mitotic figures.

As suggested, we have changed the histological validation figures. We now show higher magnification images (400x) and mitotic figures present in the tumors.

The authors should report the ethnicity of the patients from which the samples are collected in Table 1, as this could become an important confounding factor in the study of MPNSTs in the future.

Ethnicity has been described in Table 1.

In most of the histological sections shown, the grafted tumor appears to be more densely cellular than the patient sample, suggesting perhaps a loss of extracellular matrix (ECM) material. Immunohistochemical staining for collagen IV and laminin should be examined to determine if the tumor cells produce similar levels of ECM in the grafts as the in situ tumors.

We agree with the referee that the grafted tumor appears to be more densely cellular than the corresponding primary tumor; this is also observed when analyzing the HE staining. This has been observed in all of the orthoxenograft models generated by our group. However, we have discussed this with our team of pathologists and all of them believe that immunohistochemical examination of collagen IV and laminin would not help to determine the amount of ECM in the grafts and primary tumors. These proteins are the principal component of basement membrane and staining is limited to the interphase between tumor cells and stromal components. However, they are not useful as a direct measure of ECM. Nonetheless, we have obtained good results with collagen IV (not with laminin) and share them here with the referee. No differences were observed between primary and orthoxenograft tumors.
The figure below shows Col-IV IHC for the primary tumor (A) and orthoxenograft tumor (B) of MPNST-NF1-002.

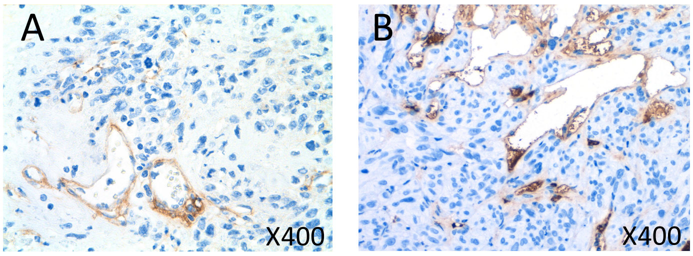

The metastatic phenotype is particularly interesting and valuable to researchers developing therapies. The authors should add photos of the late stage micrometastases to Figure S2, and make clear which models showed the metastases. Was this phenotype specific to the sporadic MPNST models? The supplemental figure is pixelated and the histology of the metastasis cannot by reviewed. A better resolution figure is needed. The metastases should also be stained for S100 to verify their MPNST origin. With the current data as presented it is not possible to exclude that these are infections in the lung.

We have investigated in more depth the presence of metastases in all of the samples collected. We present additional data and new figures illustrating our results. We have added late-stage micrometastases to Figure S2 and illustrate the models showing metastases: the two sporadic models and the model derived from the S462 cell line. The figure has a higher resolution to make it easier to analyze. For one of the metastases, we show a complete immunochemical characterization using four specific antibodies: vimentin, CD34, S100 and Ki-67.

A paragraph about metastases has been added to the Material and Methods section: "To investigate the capability of the orthoxenograft tumors to disseminate in mice, the lungs, livers and brains of 45 animals were histologically examined by H&E staining for the presence of micrometastases". This analysis was performed by an independent pathologist (August Vidal, now included in the paper as a co-author).

Moreover, the following paragraph has been added to the Results section: "To investigate distal dissemination properties, lung, brain and liver from sacrificed mice were histologically analyzed for the presence of micrometastases. Synchronic micrometastases were identified in lung from three of the models (MPNST-SP-001, MPNST-SP-002, and the orthotopically engrafted S462 cell line) (Supplementary Figure S2) but no liver or brain metastases were identified. A subgroup of orthotopically-implanted mice (the two sporadic models and MPNST-NF1-001) was kept alive for 4-6 months after tumor removal to investigate the dissemination capabilities over a longer time frame. Methachronic micrometastases were only identified in the lung from the sporadic MPNST tumors (Supplementary Figure S2)."

For the molecular analysis of SNPs, the authors analyzed samples at different times on three different Illumina chips. This has the potential for introducing batch effects that may be interpreted as differences between NF1 and sporadic tumors, or in situ tumors and xenografts. Which samples were run in which batches? Was there any common sample run on all chips to normalize batch effects? How were losses of SNPs handled between different platforms? The authors should also include the sex chromosome data and mitochondrial data where available.

We have added a supplementary table (S3) indicating the BeadChip versions used for the SNP-array analysis of the 17 samples.

Additionally:

1) SNP-array data were used basically for validation purposes, to compare the genome structure of primary tumors with their derived orthoxenografts for the different models. As such, any batch effect would introduce more differences than are really present between samples from each model, disfavoring the validation analysis.
2) As can be seen in the new Table S3, the samples were not systematically assigned to specific platforms, decreasing the possibility of batch effects between different models.
3) The SNP-array analysis was meant to provide a global view of the molecular karyotype of the samples. As such, it was not affected by the presence or absence of specific SNPs through the use of different versions of the Illumina BeadChip.
4) We have added the sex chromosomes to the CIRCOS plots. However, only autosomal chromosomes were used for validation purposes, to avoid gender-related biases. Mitochondrial data were not available for the analysis; however, since SNP-array data were used to assess the genomic structure of tumors and derived xenografts, mitochondrial data were not needed for this comparison.

For the gene expression analysis, it is not clear why the NF1-001 patient sample was not included. This would provide important information for using this model. Is sample available to include in this analysis?

Unfortunately, tumor NF1-001, received by the lab after surgery, was very small. We only had enough material to graft it to the mice and a small remaining piece for DNA extraction. There was not enough material for RNA extraction, so expression analysis could not be performed for this primary sample.

While the drug testing presented is promising, it should be noted that sorafenib and the rapamycin analog have already been tested in soft tissue sarcomas, including MPNST, without clear efficacy. In the clinical trial of sorafenib (NCT00245102), 12 MPNST patients were treated and none had a complete or partial response. In a clinical trial of temisirolimus (Okuno et al 2011 Cancer 117:3468) only 5% of sarcoma patients showed a response, and the MPNST patient in the study did not respond. While it is still possible that combinations of these therapies would be beneficial, the results shown in Figure 7 suggest that these models may not predict results in the clinic. The authors should thoroughly discuss what is known about the tested drugs in sarcoma clinical trials in the discussion section.

We completely agree with the referee. Clinical trials using inhibitors of RAS/MAPK or mTOR pathways did not appear to be very effective in patients with MPNST (Okuno et al.. 2011; Maki et al., 2009). In our models we do see a slight effect of some of these drugs in a monotherapy scheme, but this effect is not as strong as when a combination of two drugs is used. It is possible that a similar process occurs in the tested patients, hence small effects of the drugs are not easily observed. In the paper by Maki et al., three out of 12 patients showed stable disease after sorafenib treatment, similar to the effect observed in some of our models. Moreover, two of the nine patients without response experienced a certain grade of regression of metastatic disease. In relation to the unique paper using a mTOR inhibitor in a clinical trial, only one MPNST patient was included, and

although no effect was observed, the number is too low to draw any conclusion. We have added a paragraph discussing these results, together with the potential effect of using a combined treatment, to the Discussion section. Two paragraphs have been added to the Discussion section, which read as follows:

"A recent phase II clinical trial assessed the monotherapy activity of sorafenib in patients with different types of sarcomas, including 12 patients with MPNST. No objective responses were observed in nine of these patients, although two experienced a certain grade of regression of metastatic disease. Three patients showed stable disease, suggesting that the drug had only a small effect (Maki et al., 2009). Although the results of the clinical trial do not appear very promising, sorafenib should be considered in combination with other agents, particularly taking into account the preclinical model results presented in this study."

"In a recent clinical trial of temisirolimus (Okuno et al., 2011) only 5% of sarcoma patients responded to treatment, and the only patient with a MPNST did not respond. Although these are the first in-human results of this treatment for sarcomas, the numbers are too small to rule out the role of mTOR inhibitors as therapeutic agents, in particular for MPNST. As our results indicate, a combination of rapamycin and drugs targeting other pathways may be beneficial for MPNST patients."

Moreover, although our short-term drug treatments indicate that sorafenib and rapamycin as single agents could promote tumor size reduction and histological response (measured by percentage of necrosis and number of mitosis), we do not observe any complete response. In addition, when analyzing long-term response measured by tumor relapse time (new Figure 7B), we observed that combined treatments are much more effective than single-agent schemes. This is why we propose that future clinical trials should consider combined drug regimens to treat MPNST.

Throughout the manuscript the authors draw preliminary conclusions on the comparison of NF1-associated MPNSTs and sporadic MPNSTs. Given that the 2 NF1 patient derived xenografts come from the same patient, it is hard to say whether the NF1 tumors are clustering because they are genetically related, because they come from a young patient, or because they are NF1 associated. These preliminary conclusions on differences between sporadic MPNST and NF1 MPNST should be removed from the abstract, from the molecular genomic results (SNP, exomeseq, and gene expression), the entire section comparing sporadic vs NF1-associated models, and the explanation of the paper. While it is useful to point out differences between the 5 models, there are not enough samples in any one group to conclude these differences correlate to NF1 disease, the mutation in the NF1 gene, the age of the patient, or loss of linked genes on chr 17 during LOH. All of these possibilities should be included in the discussion section.

We agree with the referee that the limited number of models does not allow us to draw general conclusions regarding NF1 vs. sporadic models. Accordingly, we limited ourselves to describing the differences between models. As suggested by the referee, we have therefore removed the section "Sporadic vs. NF1-associated orthoxenograft mouse MPNST models" and adapted the content of the Abstract and the main text dealing with this comparison.

Additional details should be added to the Material and Methods section: What are the conservation protocols for the patient tumors? Please describe in more detail or cite another reference.

This aspect has been described in more detail as follows:

"After surgery, the tumor was sent to our pathology service where it was analyzed following standard protocols. Simultaneously, a piece of each tumor was stored in DMEM, 10% FBS culture medium at room temperature before being sent to our molecular unit. Once in our laboratory the tumor was divided into sections, processed and preserved in order to have material for different purposes. Small pieces of each tumor were directly frozen in liquid nitrogen so that DNA, RNA and/or protein could be obtained when needed. Small pieces were frozen in appropriate culture media so that cell culture experiments or mice engraftments could be performed. Informed consent was obtained from all subjects and the study received the approval of the IDIBELL Ethics Committee."

What committee approved the human tissue study?

"Informed consent was obtained from all subjects and the study received the approval of IDIBELL Ethics Committee." This sentence has been added to the Material and Methods section.

For the grafting into the sciatic nerve, please provide more details. Was the piece of tumor placed along side the nerve? Was the epineurium breached? Why were the S462 cells injected into the muscle and not into the sciatic nerve?

We have rewritten the description of the orthotopic implantation to clarify the procedure. A small incision was made in the muscle to display the sciatic nerve. A piece of tumor was grafted there and grown surrounding the epineurium. The key points are that the tumor is fixed to the surface of sciatic nerve with synthetic monofilament, non-absorbable polypropylene suture (Prolene 7.0), and that the epineurium was not breached. Primary tumors were grafted both orthotopically and subcutaneously in three different mice in the first passage. To establish the orthoxenograft model from the S462 NF1-MPNST cell line, we injected 0.3 ml of the cell suspension ($3x10^6$ cells) with a needle directly in the upper thigh muscle using Matrigel (a solubilized tissue basement membrane matrix rich in extracellular matrix proteins). This enabled tumor growth around the epineurium. Once the tumor had developed, it was harvested, cut into small fragments, and, like a regular orthoxenograft, anchored on the surface of sciatic nerve with Prolene 7.0. We did not try to inject cells with a fine titanium needle as previous described (Perrin et al., 2007) because our goal is to obtain a tumor from the cell line and then use this tumor by applying the same methodology as for primary tumors.

How were drug formulations chosen? Had these been used previously in preclinical mouse studies (please cite references).

The three selected drugs have been used in preclinical mouse studies, hence the same schemes were used in our models. The references are now cited in the section, as follows: "Doxorubicin dose was chosen on the basis of studies in which intraperitoneal administration at 8 mg/kg was tested in a xenograft model derived from a MPNST cell line (Johansson et al., 2008). Rapamycin administered intraperitoneally at 5 mg/kg was used previously in a genetically engineered MPNST mouse model (Johannessen et al., 2008), and the sorafenib dose was chosen on the basis of preclinical studies in which daily oral administration of Sorafenib at 30-60 mg/kg was tested in several tumor models (Wilhelm, 2004)."

Minor Issues.

Turk et al J Vis Exp. 2011 Mar 7;(49).pii: 2558. doi: 10.3791/2558 should be added to the references on pg 5 as a orthotopicxenograft generated from an MPNST cell line.

This reference has been added.

Figure 3 panels are out of order in the manuscript. Figure 3A could be moved to the bottom for Figure C and other panels moved accordingly so the figures are in the order cited in the manuscript. In the current Figure 3A the green bar and the red line are not defined in the figure legend.

Figure 3 has been reordered as suggested. The green bar and red line are now defined in the figure legend.

Pg 8, 2nd new paragraph: Change "composed by" to "composed of."

This has been changed as suggested.

Pg 10, 1st new paragraph: Change "Table SI" to "Table S1."

This has been changed as suggested.

Should supplemental figure 5 be cited here?

We believe that supplementary figure 5 should not be cited there.

Make paragraph indenting consistent throughout.

Indenting has been reviewed throughout the manuscript.

The S462 cell line was generate by Lan Kluwe's group and is described in Frahm et al Neurobiology of Disease (2004) 16:85-91 (patient 6). The citation should be added to the manuscript.

This reference has been added.

Pg 21, 3rd paragraph: Change "tight" to "thigh" in the 3rd line.

This has been changed as suggested.

Supplementary Figure S2 legend: The second line appears to be a fragmented sentence. Do the authors mean the tumors of the orthotopically implanted mice or the lungs? It doesn't make sense to say the "mice were removed by surgery." Is the histology of the middle panel of the tumor or of the lung or liver?

This figure legend has been completely rewritten as the figure was redesigned.

2nd Editorial Decision 06 February 2015

Thank you for the submission of your revised manuscript to EMBO Molecular Medicine. We have now received the enclosed reports from the referees that were asked to re-assess it. As you will see the reviewers are now globally supportive.

Reviewer 3, however, has some remaining concerns of importance. His/her comments are quite clear and I would simply like to mention a few salient ones. The Reviewer notes that you still draw comparisons between NF1 and sporadic tumours, which appears unjustified based on the numbers available. S/he also asks you to tone down your statements on transferability of the combination treatment to the clinic. Reviewer 3 also lists other items that require your action.

I am prepared to make an editorial decision on your next, final version, provided you send me a point-by-point rebuttal and accordingly modify the manuscript. Please also upload an extra copy of your manuscript with the changes highlighted. In the likely event of acceptance, I would be asking you for the following final editorial amendments, which at this point I suggest you directly incorporate in your final revision:

1) As per our Author Guidelines, the description of all reported data that includes statistical testing must state the name of the statistical test used to generate error bars and P values, the number (n) of independent experiments underlying each data point (not replicate measures of one sample), and the actual P value for each test (not merely 'significant' or 'P < 0.05').
2) We are now encouraging the publication of source data, particularly for electrophoretic gels and blots, with the aim of making primary data more accessible and transparent to the reader. Would you be willing to provide a PDF file per figure that contains the original, uncropped and unprocessed scans of all or at least the key gels used in the manuscript? The PDF files should be labeled with the appropriate figure/panel number, and should have molecular weight markers; further annotation may be useful but is not essential. The PDF files will be published online with the article as supplementary "Source Data" files. If you have any questions regarding this just contact me.
3) Every published paper now includes a 'Synopsis' to further enhance discoverability. Synopses are displayed on the journal webpage and are freely accessible to all readers. They include a short standfirst (to be written by the editor) as well as 2-5 one sentence bullet points that summarise the paper (to be written by the author). Please provide the short list of bullet points that summarise the key NEW findings. The bullet points should be designed to be complementary to the abstract - i.e. not repeat the same text. We encourage inclusion of key acronyms and quantitative information. Please use the passive voice. Please attach these in a separate file or send them by email, we will incorporate them accordingly.

Please submit your revised manuscript within two weeks. I look forward to seeing a revised form of your manuscript as soon as possible.

I look forward to reading a new revised version of your manuscript as soon as possible.

***** Reviewer's comments *****

Referee #1 (Comments on Novelty/Model System):

This study EMM-2014-04430 by Castellsague et al. describes the establishment of 5 patient-derived MPNST orthoxenograft models and preliminary results of pre-clinical pharmacological testing on these models.

Authors have addressed ours questions and provided a revised version that meets our requirements.

This is a well written study describing a preliminary study on a limited number of orthoxenografts. This study (describing an orthoxenograft model of MPNST) may be useful for the NF1 field.

Referee #2 (Comments on Novelty/Model System):

This is the first well documented xenograft model system for MPNST and is done at high quality.

Referee #2 (Remarks):

This revised manuscript describes methods for establishing malignant peripheral nerve sheath tumor orhtoxenografts. While done on a limited number of cases, this work will be valuable for the MPNST field. The results are meaningful and interpretations correct.

Referee #3 (Remarks):

In the revision by Castellsague et al, the questions raised in the prior review have been addressed. The primary impact of this work is the careful and thorough characterization of several new models for preclinical testing of drugs in vivo. The preclinical results using combination therapy with sorafenib provide a proof of concept that this model can be used for drug testing, and that sorafenib can be combined with other drugs to show a greater effect. It should be noted, however, that the effect sizes seen seem relatively modest, so it would be premature to conclude that these particular combinations would be successful in clinical trials.

There are still a few remaining issues to be resolved prior to publication:
1) In Figure 7B is the data mislabeled? The text describes sorafenib+doxorubicin as the most effective combination, but the graph clearly shows that rapamycin + doxorubicin is the best combination for longer time to relapse.
2) The authors still draw comparisons between NF1 and sporadic tumors that are inappropriate given the small number of tumors compared (page 11, 20, and 22). The differences could be entirely based on the genetic background of the NF1 patient, regardless of his having neurofibromatosis.

Minor Issues:
1) The abstract implies that graphs were injected into the sciatic nerve (line 9). This should be changed to "next to" or "adjacent." The injection of the cell line into the muscle is very different from being in the sciatic nerve. Similarly in the Results on page 7, paragraph 1, line 4, change "grafted into" to grafted onto"
2) Page 14 describes 6 treatment groups instead of 7. The distinction of oral and ip rapamycin should be added.
3) Page 16, second new paragraph, line 2, change "following the same" to following similar" - injection into the muscle is rather different than tying the tumor graft in direct contact with the sciatic nerve.
4) Page 21, line 5: I disagree that little data is available for in vivo models on sorafenib, given that a clinical trial has been conducted on MPNST patients!
5) Page 22, second paragraph, line 8, because the combination results would likely not achieve a partial response by RECIST in a clinical trial (assuming no species differences), the conclusions should be toned down (only 1 of the 5 models show a strong response of tumor shrinkage). "The most effective treatment..." should be changed to "The most effective treatment tested..." to make it clearer that more work is needed to find the optimal treatment for MPNST patients.
6) Page 28, line 5: Typo? "an" >> "and"?

7) In Figure 5 the numbers of mutations changed from the previous version. Why?
8) In table 3, are the results shown for oral or ip rapamycin? Please indicate in the table.

2nd Revision - authors' response 12 February 2015

We have addressed the issues raised by referee three and the specific Editorial comments. We hope that the paper is now suitable for publication in EMM.

Note that the comments of referee 3 and the editorial board are given in italic type.

Referee 3 comments

In the revision by Castellsague et al, the questions raised in the prior review have been addressed. The primary impact of this work is the careful and thorough characterization of several new models for preclinical testing of drugs in vivo. The preclinical results using combination therapy with sorafenib provide a proof of concept that this model can be used for drug testing, and that sorafenib can be combined with other drugs to show a greater effect. It should be noted, however, that the effect sizes seen seem relatively modest, so it would be premature to conclude that these particular combinations would be successful in clinical trials.

There are still a few remaining issues to be resolved prior to publication:

1) In Figure 7B is the data mislabeled? The text describes sorafenib+doxorubicin as the most effective combination, but the graph clearly shows that rapamycin + doxorubicin is the best combination for longer time to relapse.

The referee is right, the data were mislabeled. We have corrected the figure and are grateful for the observation.

2) The authors still draw comparisons between NF1 and sporadic tumors that are inappropriate given the small number of tumors compared (page 11, 20, and 22). The differences could be entirely based on the genetic background of the NF1 patient, regardless of his having neurofibromatosis.

We agree with the referee. In page 11 (Results section) we have removed any mention to a comparison between NF1 and sporadic MPNST. In page 20 (Discussion section) we have changed the paragraph to make clear that the limited number of models presented is too low to draw any conclusion comparing both types of MPNSTs. Following that, we have left the description of the differences among the samples analyzed, but always making clear that is only respect to these samples, and not a generalization. In page 22 (last paragraph of the discussion section) we just mention the differences among the samples studied in the present work, making clear that in order to reach any conclusion a larger number of tumors need to be analyzed.

Minor Issues:

1) The abstract implies that graphs were injected into the sciatic nerve (line 9). This should be changed to "next to" or "adjacent." The injection of the cell line into the muscle is very different from being in the sciatic nerve. Similarly in the Results on page 7, paragraph 1, line 4, change "grafted into" to grafted onto"

We have made the changes according to the referee's instructions.

2) Page 14 describes 6 treatment groups instead of 7. The distinction of oral and ip rapamycin should be added.

Referee 3 is right, the distinction has been added.

3) Page 16, second new paragraph, line 2, change "following the same" to following similar" - injection into the muscle is rather different than tying the tumor graft in direct contact with the sciatic nerve.

This has been changed as suggested.

4) Page 21, line 5: I disagree that little data is available for in vivo models on sorafenib, given that a clinical trial has been conducted on MPNST patients!

"Little" has been removed.

5) Page 22, second paragraph, line 8, because the combination results would likely not achieve a partial response by RECIST in a clinical trial (assuming no species differences), the conclusions should be toned down (only 1 of the 5 models show a strong response of tumor shrinkage). "The most effective treatment..." should be changed to "The most effective treatment tested..." to make it clearer that more work is needed to find the optimal treatment for MPNST patients.

We have rewritten the sentence as suggested.

6) Page 28, line 5: Typo? "an" >> "and"?

The referee is right. This has been corrected.

7) In Figure 5 the numbers of mutations changed from the previous version. Why?

Referee is right. In the process of resubmission we uploaded an incorrect, old file. Now the file is correct and it is the same of the previous version. Thank you for notice it.

8) In table 3, are the results shown for oral or ip rapamycin? Please indicate in the table.

Only oral administration of rapamycin was analyzed. This is now indicated in the table.

Editorial comments

1) As per our Author Guidelines, the description of all reported data that includes statistical testing must state the name of the statistical test used to generate error bars and P values, the number (n) of independent experiments underlying each data point (not replicate measures of one sample), and the actual P value for each test (not merely 'significant' or 'P < 0.05').

The names of statistical tests are given in the material and methods section as follows: "Significance was tested by the Wald test and p values were adjusted by Bonferroni correction to address the problem of multiple comparisons due to multiple testing (Supplementary Table S4)."

The list of the exact p-values for drug experiments (Figure 7A) has been added as a table in the Supplementary material file.

2) We are now encouraging the publication of source data, particularly for electrophoretic gels and blots, with the aim of making primary data more accessible and transparent to the reader. Would you be willing to provide a PDF file per figure that contains the original, uncropped and unprocessed scans of all or at least the key gels used in the manuscript? The PDF files should be labeled with the appropriate figure/panel number, and should have molecular weight markers; further annotation may be useful but is not essential. The PDF files will be published online with the article as supplementary "Source Data" files. If you have any questions regarding this just contact me.

This does not apply in our case.

3) Every published paper now includes a 'Synopsis' to further enhance discoverability. Synopses are displayed on the journal webpage and are freely accessible to all readers. They include a short standfirst (to be written by the editor) as well as 2-5 one sentence bullet points that summarise the paper (to be written by the author). Please provide the short list of bullet points that summarise the key NEW findings. The bullet points should be designed to be complementary to the abstract - i.e. not repeat the same text. We encourage inclusion of key acronyms and quantitative information. Please use the passive voice. Please attach these in a separate file or send them by email, we will incorporate them accordingly.

3rd Editorial Decision 17 February 2015

I was just about ready to accept your manuscript for publication when, while performing our pre-publishing quality control and image screening routines, we noticed issues pertaining to Fig.s 2 and 3 in your manuscript, which prevent us from moving forward with your manuscript.

Specifically,

1) Please make sure you indicate in the larger images wherefrom the insets were magnified (e.g. with a box)
2) In one case, the magnified area seems to have been inverted with respect to the original image. Please fix.

Please submit your new figures as soon as possible so that I can proceed with acceptance.

© EMBO
